# Field-deployable, quantitative, rapid identification of active Ebola virus infection in unprocessed blood†
†Electronic supplementary information (ESI) available. See DOI: 10.1039/c7sc03281a


**DOI:** 10.1039/c7sc03281a

**Published:** 2017-09-25

**Authors:** Kavit Shah, Emma Bentley, Adam Tyler, Kevin S. R. Richards, Edward Wright, Linda Easterbrook, Diane Lee, Claire Cleaver, Louise Usher, Jane E. Burton, James K. Pitman, Christine B. Bruce, David Edge, Martin Lee, Nelson Nazareth, David A. Norwood, Sterghios A. Moschos

**Affiliations:** a Westminster Genomic Services , Department of Biomedical Sciences , Faculty of Science and Technology , University of Westminster , 115 New Cavendish Str , London W1W 6UW , UK; b BGResearch Ltd. , 6 The Business Centre, Harvard Way, Harvard Industrial Estate , Kimbolton , Huntingdon PE28 0NJ , UK; c Department of Biomedical Sciences , Faculty of Science and Technology , University of Westminster , 115 New Cavendish Str , London W1W 6UW , UK; d BioGene Ltd. , 8 The Business Centre, Harvard Way, Harvard Industrial Estate , Kimbolton , Huntingdon PE28 0NJ , UK; e Public Health England , National Infection Service , High Containment Microbiology Department , Porton Down , Salisbury , Wiltshire SP4 0JG , UK; f Fluorogenics LIMITED , Building 227, Tetricus Science Park, Dstl Porton Down , Salisbury , Wiltshire SP4 0JQ , UK; g Diagnostic Systems Division and Virology Division , United States Army Medical Research Institute of Infectious Diseases , Fort Detrick , MD 21701-5011 , USA; h Department of Applied Sciences , Faculty of Health and Life Sciences , Northumbria University , C4.03 Ellison Building, Ellison Place , Newcastle Upon Tyne , Tyne and Wear NE1 8ST , UK . Email: Sterghios.moschos@northumbria.ac.uk ; Tel: +44(0) 191 215 6623

## Abstract

The West African Ebola virus outbreak underlined the importance of delivering mass diagnostic capability outside the clinical or primary care setting in effectively containing public health emergencies caused by infectious disease. Yet, to date, there is no solution for reliably deploying at the point of need the gold standard diagnostic method, real time quantitative reverse transcription polymerase chain reaction (RT-qPCR), in a laboratory infrastructure-free manner. In this proof of principle work, we demonstrate direct performance of RT-qPCR on fresh blood using far-red fluorophores to resolve fluorogenic signal inhibition and controlled, rapid freeze/thawing to achieve viral genome extraction in a single reaction chamber assay. The resulting process is entirely free of manual or automated sample pre-processing, requires no microfluidics or magnetic/mechanical sample handling and thus utilizes low cost consumables. This enables a fast, laboratory infrastructure-free, minimal risk and simple standard operating procedure suited to frontline, field use. Developing this novel approach on recombinant bacteriophage and recombinant human immunodeficiency virus (HIV; Lentivirus), we demonstrate clinical utility in symptomatic EBOV patient screening using live, infectious Filoviruses and surrogate patient samples. Moreover, we evidence assay co-linearity independent of viral particle structure that may enable viral load quantification through pre-calibration, with no loss of specificity across an 8 log-linear maximum dynamic range. The resulting quantitative rapid identification (QuRapID) molecular diagnostic platform, openly accessible for assay development, meets the requirements of resource-limited countries and provides a fast response solution for mass public health screening against emerging biosecurity threats.

## Abstract 2

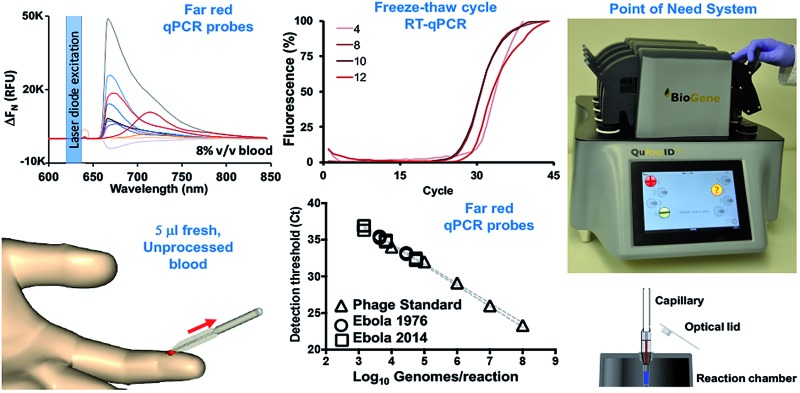
The West African Ebola virus outbreak underlined the importance of delivering mass diagnostic capability outside the clinical or primary care setting.

## Introduction

The 2013–2016 Ebola virus (EBOV) outbreak resulted in over 28 500 confirmed cases across three continents with an overall mortality rate of 39.5% (http://www.who.int/mediacentre/news/releases/2016/ebola-zero-liberia/en/). Unlike previous outbreaks, lack of preparedness in West Africa, local customs that facilitated transmission, and the generally poor healthcare infrastructure were key to the unprecedented size of the outbreak. An international emergency response was therefore necessary to arrest what rapidly became a global health and security threat. Thus, at the peak of transmission in August 2014, the World Health Organization (WHO) declared the outbreak a public health emergency of international concern (PHEIC; ; http://www.who.int/mediacentre/news/statements/2014/ebola-20140808/en/). It quickly became clear that success in containing the outbreak would pivot on three actions: (1) reliable diagnosis of suspected/probable cases *i.e.* symptomatic individuals; (2) quick isolation of patients confirmed as infected with EBOV, and; (3) effective contact tracing.^1^

Unfortunately, symptomatic, suspected cases were difficult to differentially diagnose against other causative agents of hemorrhagic fever, as symptoms were generally non-specific and common to other endemic, high incidence infections such as malaria, yellow fever, and Lassa fever. Furthermore, epidemiological information, where available, only marginally contributed to patient triage by increasing EBOV disease risk potential from ‘suspected’ to ‘probable’. Thus, it became essential to obtain explicit, clinical diagnostic grade evidence of active EBOV infection as quickly as possible after patient presentation. Nucleic acid amplification testing (NAAT) using thermal cycling, fluorescent probes and real time, quantitative reverse transcription polymerase chain reaction (RT-qPCR) was quickly recognized as the most reliable clinical diagnostic solution for symptomatic EBOV patients.^2^ Thus, studies during previous outbreaks^3^–^5^ and data emerging from West Africa^4^ reaffirmed that patients would present to triage only when symptoms became intense, corresponding to high viraemia levels ranging 10^5^–10^10^ genome equivalents per ml (GE per ml) of whole blood.

Importantly, RT-qPCR is an internationally adopted *in vitro* diagnostic (IVD) technology considered robust, sensitive, and highly specific. Unlike most other territories, in the USA the Food and Drug Administration (FDA) presently regulates stringently both IVD thermal cycler instruments and associated IVD assay kits through extensive clinical trials before issuing marketing authorization. Furthermore, diagnostic services based on NAAT are provided only through FDA-regulated laboratories; the services also cover infectious disease agent diagnostics, including a multitude of RNA viruses.^6^ This regulatory framework applies whether the diagnostic test is available as an independently marketed IVD kit or an assay developed in-house in the regulated laboratory using regulated reagents. Such measures enhance diagnostic service reliability, a requirement important in the context of highly infectious disease such as hazard group 3 and 4 pathogens.

Implementation of NAAT diagnostics for EBOV disease was spearheaded by the August 2014 FDA Emergency Use Authorization (EUA) of the EZ1 Taqman® assay which uses FAM-labelled fluorescent probes for RT-qPCR undertaken on one of several FDA-approved thermal cyclers (http://www.fda.gov/downloads/MedicalDevices/Safety/EmergencySituations/UCM418799.pdf). This molecular test involves the quantitative detection of two RNA substrates: the EBOV glycoprotein (GP)^7^ gene encoded in the negative RNA genome of EBOV, and the human ribonuclease P (RP) transcript which is found at high levels in human biofluid samples, as an endogenous/process control.^8^ To date, this so-called Trombley assay^7^ remains the gold standard of diagnostic care^7^,^9^,^10^ used by public health agencies worldwide and is reported to be the most sensitive and specific for semi-quantitative EBOV diagnosis in viraemic, *i.e.* symptomatic patients.^10^ Thus, the Trombley assay features a 0.0001 plaque forming unit per reaction (PFU per reaction) lower limit of detection (LLOD), across a 5 log-linear dynamic range (LDR),^7^,^9^ and analytical specificity against human RNA, and 75 other pathogens (; http://www.fda.gov/downloads/MedicalDevices/Safety/EmergencySituations/UCM408334.pdf). Of note, the qualification and performance data of this assay do not pertain to the reaction substrate, the EBOV RNA genome, but rather the amount of live virus in the sample. This relates to the use of infectious virus quantification methods in classical virology (PFU quantification assays),^11^ which are medically valuable in informing the risk of infection/transmission, rather than the biochemically relevant assay substrate concentration reported by NAAT. Yet studies with *in vitro* produced virus^9^,^12^ and macaque infection models^13^ indicate the viral genomes might be 10-fold^12^ to 10 000-fold^9^ higher than infectious virions in a given sample. This has been evidenced across many hazard group 3 and 4 viruses, and has been associated to the extent of virus passage *in vitro*.^13^ Clinical data remains limited given the preference for immediate virus inactivation for health and safety reasons.

Unfortunately, the entire Trombley procedure is slow, laboratory-based, and has considerable safety implications. Thus, venous blood is the biological sample of choice, but the large volumes needed (3.5 ml) increase the risk of uncontrolled bleeding for patients exhibiting hemorrhagic symptoms. The samples also present a risk of infection to the healthcare staff collecting them and the diagnostic laboratory personnel analyzing them, making extensive safety training essential in reducing transmission. Notwithstanding safety concerns, the Trombley assay procedure further necessitates considerable technical expertise involving multiple manual steps operating expensive laboratory infrastructure and mains-powered instrumentation, using expensive consumables stored constantly at 4 °C. Appropriately equipped facilities were scant in West Africa and the technology was not suited to laboratory-free field deployment in rural regions where need was at its highest.^2^ In addition, at ∼US$100, the cost per patient^14^ exceeded by up to tenfold the annual, *per capita* healthcare expenditure in the region.^15^ Moreover, the time-to-results, at best 5–8 hours from sample receipt, restricted throughput capacity and presented additional infection risks to EBOV-free patients awaiting results whilst in triage. Overall, at the peak of transmission, assay complexity, cost, and lack of speed to molecular diagnosis hampered the efforts to stave off transmission.

To address these challenges many industrial and academic groups responded to calls for solutions in line with the WHO ASSURED criteria^16^ relevant to resource limited settings. Thus, the ideal diagnostic test should be affordable, sensitive, specific, user-friendly, robust, rapid, equipment-free and deliverable to those in need – *i.e.* usable in the field with minimal requirements for additional resources. Ideally, this would also involve minimal biofluid volumes, such as microliter drops of blood analysed *in situ* by digital venipuncture, not dissimilar to over-the-counter, point-of-need glucometers. To date, the most clinically advanced, close-to-patient NAAT IVDs still require powered instrumentation, a fully equipped laboratory setting (fixed or mobile), training associated to multiple handling steps starting with 3.5 ml of venous blood, and consumables still far too costly for the countries most affected by such tropical disease.^2^,^10^,^17^

Here, we report our efforts to address the need for quantitative, rapid and reliable, molecular diagnosis of EBOV disease in symptomatic patients arriving at triage, under the 12 month Emergency Call Research for Health in Humanitarian Crisis (r2hc) program of the Enhancing Learning and Research for Humanitarian Assistance (ELRHA) organization. Taking into account reports that quantitative PCR (qPCR) is possible in reactions containing blood,^18^,^19^ we present evidence that this extends to RT-qPCR retaining quantitative diagnostic value without the need for substantial formulation/enzyme modifications, provided far-red dyes are used to overcome the fluorogenic inhibition effect of blood and the minimal impact on sensitivity remains clinically acceptable. We further evaluate the utility of controlled freezing and thawing cycles in disrupting virion structures to release viral genomes directly in blood samples as an energy and engineering-efficient means of eliminating manual or automated RNA extraction and purification. We further present the development of an instrument and computational approach compatible with parallel detection of multiple far-red dyes, engineered for field deployment at the point of need, in a laboratory infrastructure-free manner, and with minimal end-user training requirements. Finally, we determine the performance of this approach in detecting and quantifying EBOV against the gold standard, laboratory-based Trombley assay, within the context of screening symptomatic patients in a triage setting.

## Results

### Benchmark assay system performance

Given the emerging evidence that the Trombley GP assay performed better against a number of other RT-qPCR assays for EBOV,^7^,^9^,^10^ and to focus our efforts, we adopted the Trombley assays for the EBOV GP and NP genes in our work. Moreover, to overcome limited biosafety level 4 (BSL4) facility and staff availability due to increased screening services demand, we used surrogate EBOV templates. We reasoned against chemically synthesized RNA as a template because blood nucleases rapidly degrade such biomaterials at ambient temperatures,^20^ which could prove problematic with minimal assay formulations. On the other hand, *de novo* construction of *in vitro* produced, non-infectious, and/or replication-deficient EBOV virions,^21^ an approach common for many well-studied species of mammalian viruses, was actively discouraged outside high containment for reasons of health, safety, and biosecurity. We thus elected to use commercially available recombinant MS2 coliphage as our primary surrogate template (AR14), despite the robustness of this virion compared to the easily fragmentable nature of filovirus particles,^22^ and pseudotyped lentivirus (PV) based on HIV manufactured in-house as a fallback template (see ESI, Fig. S1†).^23^

**Fig. 1:**
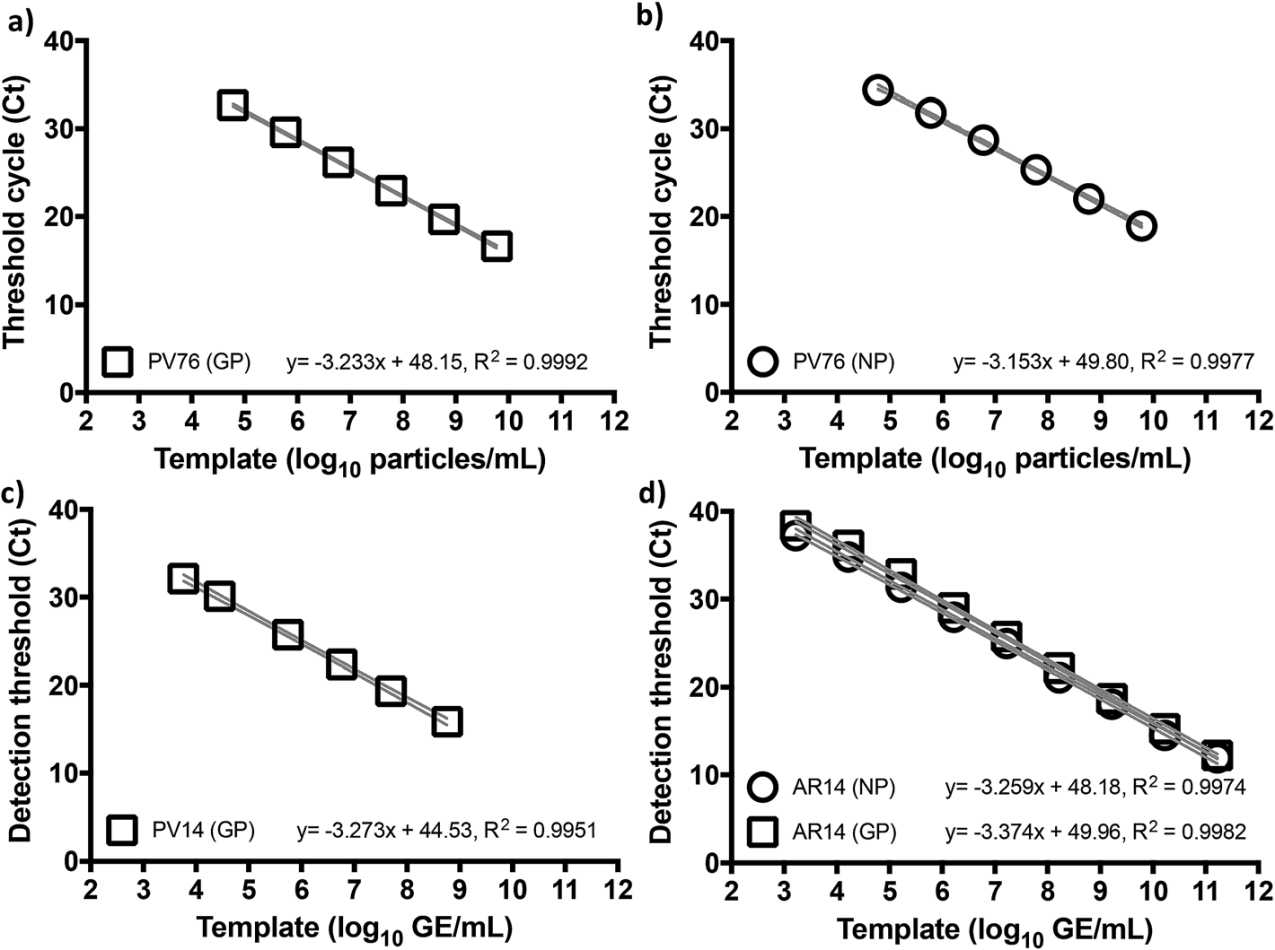
Trombley qRT-PCR assay performance is not affected by Ebola virus genetic drift. Serially diluted RNA extracts of HIV-1 pseudotyped Lentiviruses encoding the 1976 (a, b) and 2014 (c) assay target sequences (PV76 and PV14, respectively) for the Trombley (a, c) glycoprotein (GP) and (b, d) nucleoprotein (NP) assays were subjected to one-step qRT-PCR on an ABI7500 using the EZ1 SOP. These tests were repeated on RNA extracts of the AR14 template (d) to demonstrate an 8 log LDR with independent curve fits per assay (<0.01% probability of single curve fit, Akaike's Informative Criteria test). Means ± 95% confidence bands are shown.

To benchmark Trombley assay performance with these standards, we performed the plate-based EZ1 kit standard operating procedure (SOP) on an ABI7500 real time thermal cycler.^7^ We thus used healthy volunteer blood freshly collected by superficial venipuncture, spiked with AR14 to a final concentration of 1.67 × 10^11^ GE per ml of blood. Viral RNA was then extracted using the QIAamp Viral RNA Mini Kit (Qiagen, Manchester, UK), purified extracts were serially diluted with nuclease-free water and 5 μl volumes were assayed in triplicate. The results demonstrated independent amplification efficiencies for the GP and NP assays (98.0% and 102%, respectively) despite the use of the same template, resulting in distinct curve fits as determined by Akaike's informative criteria test (Fig. 1). The high concentration of AR14, however, allowed us to demonstrate a linear dynamic range (LDR) for both assays 3 logs higher than that reported previously.^7^ Moreover, the 6.67 genome equivalent per reaction (GE per reaction) lower limit of detection (LLOD) and low variability between technical replicates, as demonstrated through 95% confidence bands across the dilution series (% CV < 2.09% for GP and < 3.41% for NP), set performance standards for forward work. Furthermore, after generating PV constructs containing the Trombley assay target sequences corresponding to the 1976 EBOV Yambuka-Ecran reference genome (PV76) and the 2014 EBOV Guinea Makona reference genome (PV14), we confirmed that the single nucleotide polymorphisms identified within the West African outbreak isolate^24^ had limited impact on diagnostic assay reliability (see ESI, Fig. S1, Tables S1 and S2†).

### RT-qPCR proceeds but is fluorogenically inhibited by blood not treated with anticoagulants

**Fig. 2:**
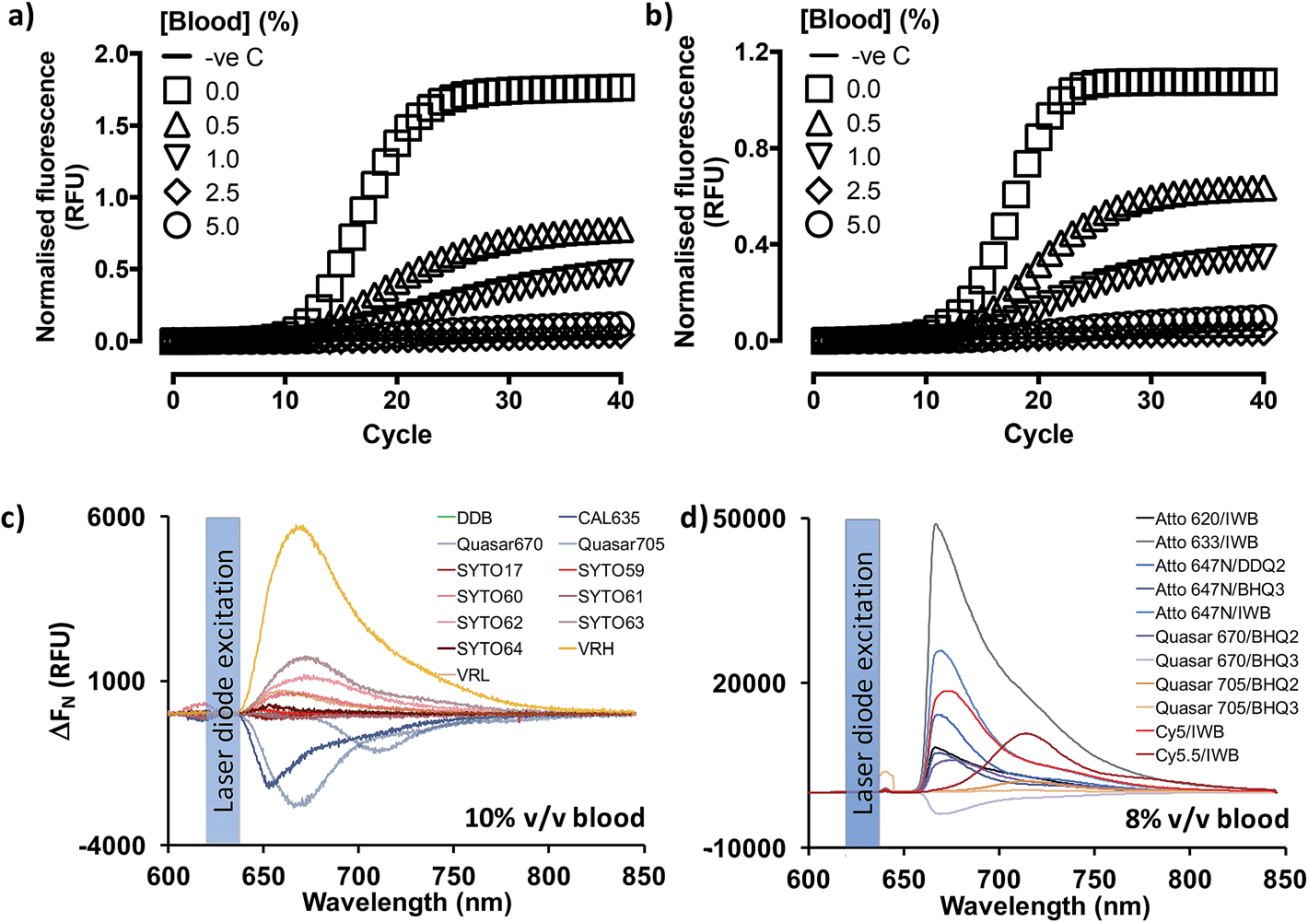
Overcoming the dose-dependent inhibitory effect of blood on qRT-PCR. Increasing amounts of fresh, unprocessed human blood introduced into qRT-PCR reactions (0.0–5.0% v/v final blood concentration) result in comparable dose-dependent inhibition of FAM-labelled Taqman® probe-based RT-PCR fluorogenicity (amplification plateau) whether the Promega hot-start wild type Taq polymerase (a) or the blood-optimised OmniTaq 2 polymerase (b) is used in conjunction with an MMuLV reverse transcriptase to detect 4 × 10^8^ copies of MS2 coliphage RNA. Reaction progression is reported for each of two technical replicates in relative fluorescence units (RFU) normalised to no template control (–ve c) against PCR cycle value. Far red/infrared fluorescent dyes (c) and dye–quencher combinations (d) were screened by spectrophotometry under 620 nm laser diode simulation for utility in blood-containing reactions. The impact of blood on dye fluorogenicity is expressed as the change (Δ*F*_N_) in background-subtracted fluorescence emission (RFU) in the presence and absence of human blood (mean of two technical replicates). DDB: deep dark blue; VRH: Viridian Red High; VRL: Viridian Red Low; IWB: Iowa Black; BHQ: Black Hole Quencher.

Previous reports^18^,^19^,^25^ indicated that PCR containing whole blood may proceed in the context of enabling mutations in the Taq DNA polymerase (OmniTaq mutant)^18^,^19^ or with at least one commercially available, research-grade polymerase supplemented with reaction enhancers.^19^,^25^ However, implementation reliability concerns had been independently expressed.^25^ Importantly, qPCR with SYBR Green I nucleic acid stain (520 nm emission peak) was only achieved with their OmniTaq enzyme^18^ and by overcoming the inhibitory effect of blood on fluorogenicity dose-dependently, *e.g.* by using 64× higher concentrations of dye for a reaction spiked with 10% v/v blood. Notably, this work involved blood and plasma treated with anti-coagulants which are known PCR inhibitors, and samples were freeze–thawed up to five times. Use of green dye-labelled Taqman® probes (CAL Fluor 540 nm) was later shown by the same group to require a complex PCR enhancer cocktail to achieve detection of as little as 7 genome equivalents per microliter (GE per μl) of DNA template with their OmniTaq polymerase in the presence of blood. Although the authors demonstrated that the cocktail could be useful for other polymerases in end-point PCR assays, they did not discuss its' use in RT-qPCR. Encouraged by these reports, and working in parallel to constructing the PV standards, we investigated whether the blood compatibility of PCR could be extended to RNA templates and RT-PCR using similar, well-characterized assays.^6^,^27^ However, in line with our projected application of analyzing patient blood immediately upon collection, we dispensed with anticoagulants and blood storage altogether. Instead, we spiked fresh human blood directly into reactions by obtaining it through digital venipuncture *ad hoc*. We thus assembled one-step RT-qPCR reactions with a commercially available Taq polymerase (GoTaq® G2 Flex; Promega, Inc) or the blood-tolerant OmniTaq 2 (DNA Polymerase Technology, Inc.), using the manufacturer's mastermix, respectively, and 5 U per reaction of GoScript® Moloney Murine Leukemia virus reverse transcriptase (Promega). Importantly, although these mastermix formulations remained proprietary, both suppliers confirmed that neither formulation featured additives to overcome PCR inhibitors. Working on wet ice, these reactions were spiked with 4 × 10^8^ copies of *Escherichia coli* MS2 phage RNA, a FAM-labelled Taqman® probe (518 nm emission peak), and increasing concentrations of fresh blood (Fig. 2). To our surprise, our results indicated that RT-PCR successfully proceeded in the presence of blood with no obvious differences between the hot-start, wild type Taq polymerase (Fig. 2a) and the blood-adapted OmniTaq mutant (Fig. 2b). However, in both cases, blood inhibited the fluorogenic signal of the probe in a concentration-dependent manner.

To determine whether this was a limitation of using fluorescently labelled probe detection chemistry, we evaluated qPCR output with OmniTaq 2 against 5.5 × 10^3^ copies of lambda phage DNA, with a 10× higher Sybr Green I concentration and up to 5% v/v blood, in line with the stain/blood ratios indicated by Kermekchiev *et al.* As with the FAM-labelled probe, this reaction format also exhibited blood concentration-dependent inhibition of fluorogenic signal (Fig. S2†). Taken together, these results suggested that qPCR and RT-qPCR might be achievable in the presence of blood despite the inhibitory effect of heme,^25^ with wild type DNA polymerases in minimal mastermix formulations, provided no anticoagulants were used during blood sampling, and the inhibitory effect of blood on dye fluorogenicity could be overcome.

### Far-red probe dyes and DNA stains enable RT-qPCR in blood

Given the spectral properties of blood and the better signal-to-noise ratio of far-red dyes in other optical platforms such as confocal microscopy and *in vivo* bioimaging,^26^ we hypothesized far red/infrared fluorophores might resolve the fluorogenic inhibition effect of blood. To examine this hypothesis, the emission spectra of a gamut of commercially available, far-red nucleic acid stains (SYTO® family) and fluorescent dyes (CAL635, Quasar 670, Quasar 705) was assessed in the presence or absence of fresh human blood (Fig. 2c). Using an Ocean Optics Maya LSL fluorimeter, we also took the opportunity in these experiments to evaluate a variety of light sources: light emitting diodes (Luxeon Phillips and Dragon Osram), filtered white light sources and laser diodes. In these studies, use of a laser diode source provided power output advantages that were paralleled by several reduced engineering problems, such as optical fiber coupling and structural stability, and dispensation of wavelength filtering requirements because lasers are a discrete wavelength light source. Thus, use of this source provided more excitation power to the vessel and, with temperature stabilization, a more stable power and wavelength that any of the power LEDs tested.

These studies also suggested that the emission peaks of far red DNA stains were not consistently affected, with some exhibiting minimal inhibition (*e.g.* 13% for SYTO-17), and others over resulting in 700% signal increase (SYTO-63), probably on account of the differential cell permeability and individual binding properties of these stains to extracellular/intracellular DNA and RNA (Fig. S3†). Thus, whilst SYTO-59 (ref. 27) and SYTO-16 (ref. 28) are cell-permeable DNA stains used for leukocyte labelling in flow cytometry, SYTO-62 (ref. 29) appears more sensitive to apoptotic cells. However, its selectivity between DNA and RNA remains unclear. Moreover, SYTO-59 (ref. 30 and 31) and SYTO-61 (ref. 30) effectively enter erythrocytes for the selective detection of *Plasmodium falciparum* infection, and SYTO-64 (ref. 32) can selectively stain nucleated erythroid precursor cells, suggesting SYTO-59, -61 and -64 might be more selective for DNA over RNA. In our whole blood assay format, however, SYTO-17 and SYTO-61 showed comparable levels of peak signal loss, with SYTO-60, -62, and -63 exhibiting a substantial increase in peak signal levels, followed by SYTO-59 and SYTO-64 (<20% peak signal increase). These reports and our results therefore indicate SYTO-59, and -64 might have direct utility in substituting SYBR Green I on whole blood qPCR and RT-qPCR, especially since SYTO-64 has been previously demonstrated to have no impact on PCR efficiency.^33^ In stark contrast to the nucleic acid stains, the probe-compatible Quasar 670, Quasar 705 and CAL635 dyes consistently exhibited some 30–50% signal inhibition (Fig. 2c). However, all three allowed for 15.2 dB, 9.98 dB and 15.9 dB signal-to-noise ratios at their corresponding emission peaks (Fig. S3A–C†), respectively, relative to background blood emission spectra (Fig. S3L†). Taken together, these results indicated that the fluorogenic inhibition effect of blood might be restricted to green dyes and perhaps even yellow-orange dyes, making far red fluorophores in general appropriate substitutes for qPCR and RT-qPCR.

We proceeded to investigate this by procuring the EZ1 assay GP probe conjugated to an array of far-red dye/quencher combinations, in the presence of 8% v/v fresh human blood (Fig. 2d and S4†). Using a custom optics array we confirmed adequately detectable signal emission with all dye/quencher combinations, although some data suggested quencher-specific interactions with the biological matrix similar to other reports involving cell^34^ and animal^35^ assays (see ESI†). With signal nonetheless detected with all dyes, we triaged dye/quencher pairs based on manufacturing cost, probe yield/purification, and signal strength to arrive at Quasar 670/BHQ2 as the optimal substitute chemistry, particularly since stability issues were also experienced with BHQ3 probes between batches.

**Fig. 3:**
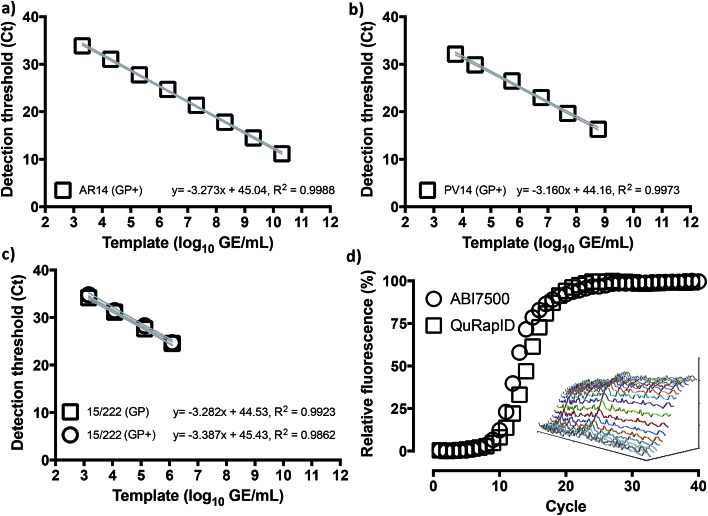
Assay migration to the blood-compatible Trombley+ chemistry has no impact on assay performance. Triplicate ABI7500 reactions with the Trombley+ GP assay chemistry (GP+) on 10-fold serial dilutions of RNA extracted from AR14 EBOV surrogate (a), PV14 EBOV surrogate (b) and National Institute of Biological Standards and Controls (NIBSC) Ebola virus NAAT standard (c) suggest retention of the template concentration-dependent 8 log-linear dynamic range and <10 genome equivalent per reaction LLOD. To compare instrument performance (d), blood-free 20 μl (ABI7500) and 62.5 μl reactions (QuRapID) on AR14 RNA extracts were carried out in parallel, demonstrating comparable reaction kinetics and Ct (exponential onset) values. Means ± 95% confidence bands are shown in (a–c). Individual replicates are shown in (d), with the inset spectrophotometric traces demonstrating example amplification spectra (*Y* axis, 5500–10 500 AFU) obtained across 665–675 nm (*X* axis) at each cycle (*Z* axis; depth) in the QuRapID reactions, with coloured lines corresponding to spectra across cycles 10–22.

We thus proceeded to examine whether use of this fluorophore/quencher combination with the Trombley GP assay (henceforth referred to as Trombley+ or GP+ assay; Table S3†) would affect RT-qPCR reaction performance. Using RNA extracts of AR14 (Fig. 3a), an analytical LLOD of 2 GE/reaction, an 8 log LDR and a % CV < 2.73% was determined for the GP+ assay, with an 103% reaction efficiency and 91.6% probability of single curve fit against the FAM-labelled GP assay (Fig. 1) using Akaike's Informative Criterion test. Importantly, these findings were reaffirmed by extending this analysis to PV14 (5.76 × 10^3^ GE per ml LLOD; % CV < 1.26; 107% efficiency; 93.0% probability of single curve fit; Fig. 3b) and external surrogate standards for EBOV NAATs^36^ (1.46 × 10^3^ GE per ml LLOD; % CV < 2.81; 97.4% reaction efficiency; 94.9% probability of single curve fit Fig. 3c). Together, these results indicated that substitution of green probe chemistries with blood compatible, far-red dye/quencher pairs such as Quasar670/BHQ2 may facilitate rapid transition of pre-validated, probe-based assays to whole blood-compatible RT-qPCR.

### Spectrophotometric signal detection for blood-containing qPCR and RT-qPCR reactions

Individual assay performance notwithstanding, clinical diagnostic reliability pivots on the elimination, if possible, of false positive/negative results. Plate-based NAATs such as the EZ1 assay minimize this risk by performing diagnostic reactions parallel to sample control reactions; separate positive and negative control reactions serve as process controls. Ensuring comparable levels of diagnostic reliability with point of care systems requires either multiplexed analysis for appropriate controls within a single reaction chamber, or microfluidic/multiple reaction chamber detection of distinct templates.

We reasoned that a single reaction chamber solution would maintain minimal consumable costs for an ASSURED-compatible point of need system. Where DNA intercalator stains such as SYBR Green I or SYTO-64 are used in a multiplexed assay approach, high resolution melting curve analysis can report individual amplicon generation.^37^ On the other hand, implementation of this methodology requires either substantial, expert end-user input,^38^ significant algorithm training^39^,^40^ and/or high complexity, costly instrumentation.^40^ Moreover, our immediately relevant EBOV GP and RP assays in the EZ1 kit involved probe-based detection. In these cases, existing assays can be readily multiplexed if no competitive PCR inhibition is experimentally evidenced, and fluorophores with spectrally distinct emission peaks are used.^41^ From an instrument perspective, probe multiplexing requires either multiple detectors, each equipped with its own detection range filter, or a single detector mounted with a moving filter wheel equipped with the relevant wavelength filters. Both solutions, however, bear manufacturing cost and instrument reliability implications due to the requirement for multiple and/or moving parts, which are ill-suited to field deployment in resource-limited countries. This makes a single detector approach lacking filtering requirements, such as that proposed herein, a valuable solution. Although our modelling efforts confirmed that mathematical^42^,^43^ spectral unmixing allowed for concomitant use of Quasar 670, Quasar 705 (duplex) and CAL635 (triplex; see ESI†), multiplexing of the GP, NP and RP assays resulted in loss of sensitivity. We therefore focused on demonstrating robust proof of principle for the platform on a singleplex format and opted for defining key multiplexing parameters through computational modelling based on the highly reliable^44^ kinetic spectral ratiometry^45^ method and the principles of the 2^–ΔΔ*C*_t_^ approach^46^ (see ESI, Fig. S5†).

**Fig. 4:**
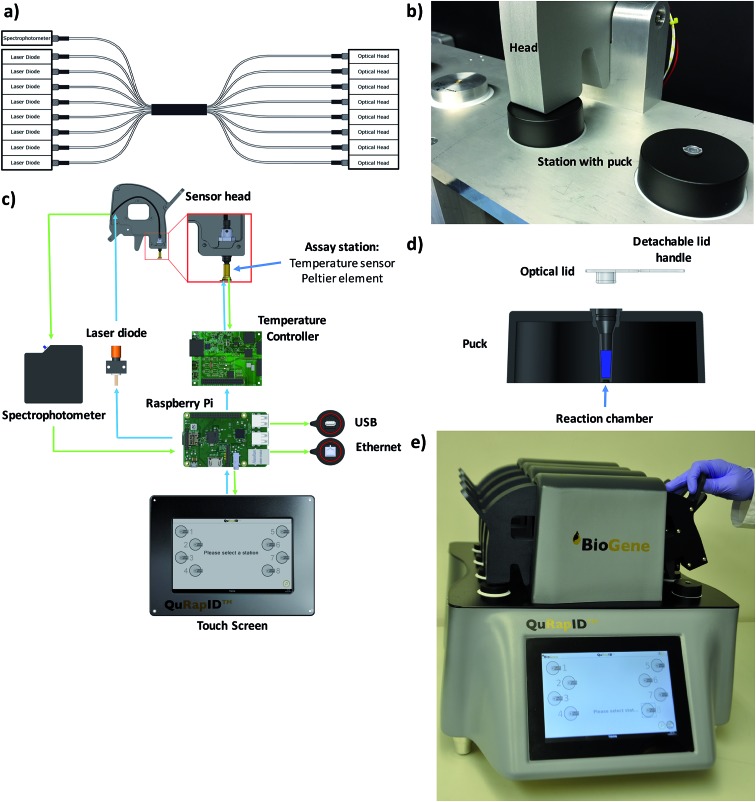
The QuRapID platform. A custom octofurcated fibre shares a single spectrophotometer end across 8 separate laser diodes and 8 separate optical heads (a) to allow for medium throughput through 8 independently operated, random access workstations (b). The workstation contains the Peltier assay station connected to a temperature controller operated by a Raspberry Pi, which is connected to the laser diodes for light emission and the spectrophotometer for data acquisition and processing, whilst being operated by a touch screen compatible with personal protective gloves (c). Each assay station accepts a single, custom fabricated, disposable puck sealed with a custom optical lid designed for easy handling (d). The resulting 20 kg, QuRapID instrument is a tabletop-sized device powered by a car alternator, car battery or mains electricity featuring eight independently operated assay stations, with software designed for simple (video online) end-user interaction in full personal protective equipment, suited for field deployment in a ruggedized case and medium throughput testing in a laboratory infrastructure-free manner (e).

These modelling results justified the use of spectrophotometry in data acquisition for a blood-compatible point of care instrument, as it would support both DNA stain high resolution melting analysis assays and quantitative, multiplexed probe-based assays. Consequently, our final excitation/emission detection array was determined to involve an Ocean Optics Maya LSL tuned to detect all light in the range 600–800 nm. To offer the capacity for parallel, independent sample testing, the instrument was also configured to offer 8 independent sample wells; this necessitated a custom octofurcated fiber (Fig. 4a) with a shared spectrophotometer end consisting of 8 × 0.22 NA, low OH, 200 μm cored fiber terminating in a single keyed SMA connector attached to the spectrophotometer. In order to allow random access and to facilitate the use of a single shared spectrophotometer, each well was illuminated individually so that any spectra observed could be attributed to a specific sample. The emission/collection legs of the fibers terminated at one end at an individual 635 nm laser diode per reaction position and the other located above the reaction vessel itself, into a barrel lens for focusing and collimating the excitation emission (Fig. 4b and c). The laser diode was built into a housing with an SMA connector and 650 nm bandpass built in to remove any excitation outside of the 635 nm laser line (FB650-40, Thorlabs). A 650 nm longpass filter was built into the entrance of the MAYA LSL to further filter unwanted laser light from the resulting emission spectra.

To confirm that implementation of this emission/detection chemistry and optics array would perform as expected, we first built a single well, Peltier-based thermal cycling testing rig (Fig. 4b) around our spectrophotometric testing array. We also mold-manufactured a custom designed reaction consumable, molded from a thermally conductive (15 W m^–1^ K^–1^) carbon loaded polypropylene compound consisting of 56% carbon (Lati SpA, Vedano Olona, Italy) with an easy handling puck and corresponding clear optical lid (Fig. 4d) that reduced the thermal energy requirements for freezing and rapid thermal cycling. The vessel had an optically clear cap and top-down excitation/emission measurement using the previously described reflectance probe.

We next proceeded to test the amplification efficiency for the blood-compatible Trombley+ assay (Quasar 670/BHQ2 GP probe) on AR14 RNA extracts across the diagnostic laboratory standard ABI7500 instrument and consumables, *vs.* our testing rig (Fig. 4b). As the ABI7500 uses onset of amplification threshold cycle (Ct) calling as well as curve smoothening algorithms,^47^ we developed a live Ct calling protocol based on real time data processing using the raw fluorescence values obtained during the run. Data was first baselined by averaging the fluorescence values observed in the 5 cycles prior to any fluorescence rise. These new values were then screened to determine the highest fluorescence observed in each channel and a relative fluorescence value for each well was determined by dividing each cycles' observed fluorescence values by this maximum value and a graph of cycle against relative fluorescence on a 0–100 scale was plotted. The cycle number at which a 10% increase in fluorescence over baseline was taken as the Ct. The results indicated comparable reaction rates and Ct values across the two platforms (Ct 9.50 and 10.5, respectively; Fig. 3d), despite the markedly different reaction volumes, consumable thermal properties, ramping rates, instrument optics and signal processing algorithms, supporting further use of this real time thermal cycling system.

### Single chamber, phase transition, blood-compatible RT-qPCR with no pre-processing

Phase transition, such as ice crystal formation during freezing, can release intracellular contents by disrupting cytosolic and nuclear membranes. Protein denaturation at high temperatures can also disrupt ribonucleoprotein complexes, thereby releasing nucleic acids for analytical assays. Used together, it has been proposed that the two processes might enable access to bacterial DNA templates for real time PCR in a single chamber system without the need for any biological sample pre-processing (WO/2011/157989). We hypothesized that these processes would also stress viral particle structures adequately to allow for RT-PCR detection of viral RNA in a single reaction chamber.

We sought to test the hypothesis with MS2 coliphage virions (Armored RNA®), reasoning that proof of principle on such a robust template would likely extend to lipid-enveloped viruses and the more fragile Filoviruses.^22^ Manual preliminary experiments suggested ∼10 cycles of freezing and 95 °C denaturation were optimal for releasing RNA from MS2 virions, but that the high levels of RNase activity in biofluids between ambient temperatures and 85 °C (ref. 48 and 49) should be avoided (see ESI; Fig. S6†). We therefore decided to carry out further experiments using automated thermal cycling capable of reaching –20 °C, and replaced 95 °C denaturation with simple thawing. This would also allow us to further examine whether denaturation was indeed essential in releasing viral RNA genomes from MS2 virions, for them to be accessed by reverse transcriptase.

**Fig. 5:**
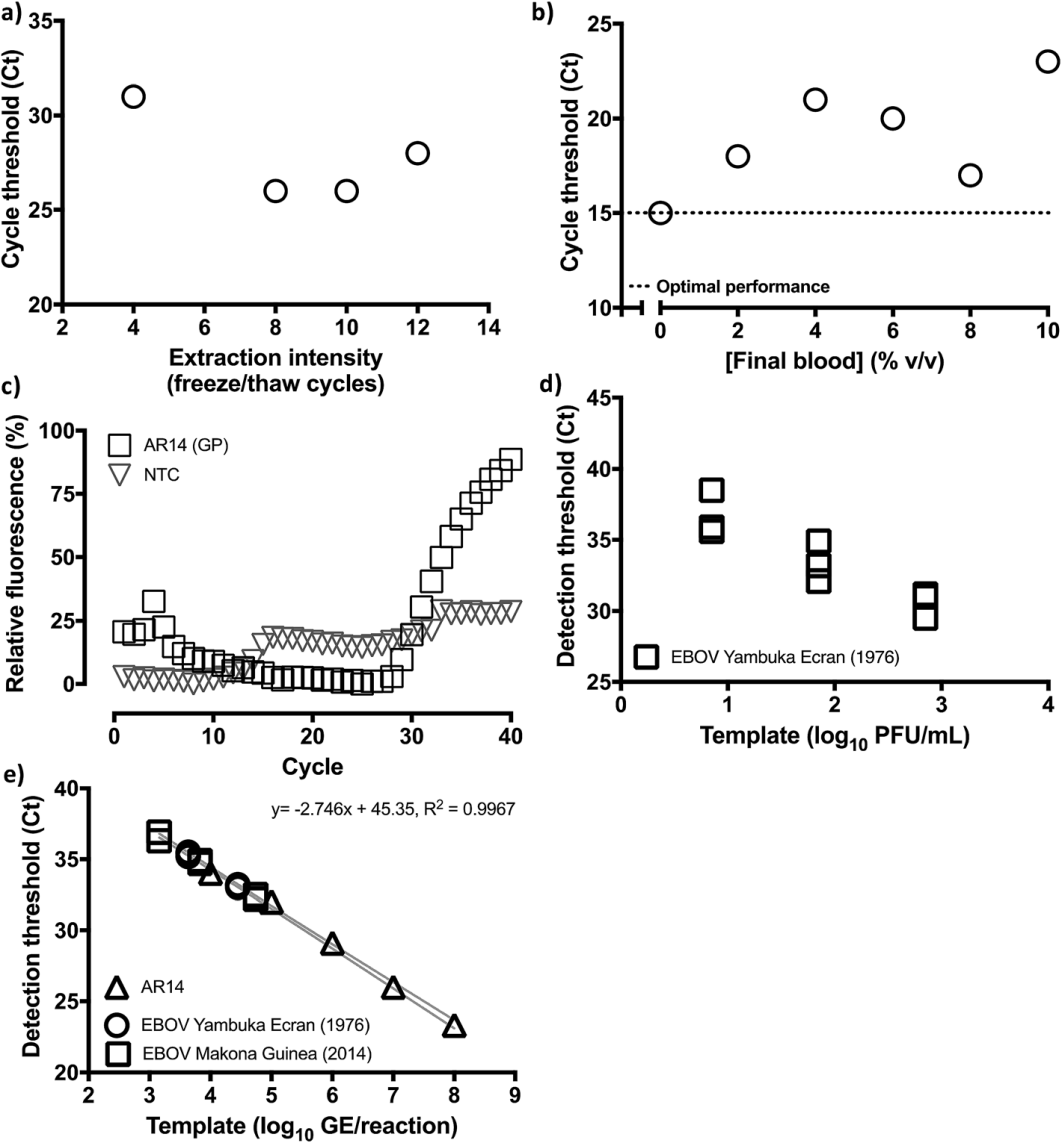
Optimised, controlled freeze–thawing of RNA viruses in probe-based qRT-PCR containing blood or serum. (a) Optimal freeze–thaw extraction of RNA from viruses within qRT-PCR master mixes in the same reaction vessel is achieved with 8–10 freeze–thaw cycles as determined through threshold cycle (Ct) detection of template amplification using the Trombley+ GP assay against 10^5^ GE per reaction of AR14 templates. No blood was used in these reactions. (b) Increasing amounts of blood spiked into reactions containing 6.6 × 10^8^ GE per reaction AR14 subjected to 10 automated freeze–thaw cycles results in a 2 Ct drop in GP Trombley+ assay performance (4×) against reactions containing no blood. The Ct call in the absence of blood in the reaction (0% v/v final blood concentration) is used as an optimal performance reference. (c) Amplification discrimination for the Trombley+ assay between no template control (NTC) reactions, and reactions containing 66 GE of AR14. Reactions were spiked with 5 microliters of human blood (8% v/v final blood concentration in the reaction) and viral genomes were extracted in the qRT-PCR blood-containing mix using 8 freeze–thaw cycles. (d) Ten-fold serial dilutions in tissue culture media of tissue cultured derived, 700 PFU per ml Ebola virus were tested in three independent reactions using the Trombley+ GP assay. Fetal bovine serum was spiked into these reactions as a fresh patient sample matrix surrogate, to an 8% v/v final concentration. (e) Calibration of Ebola virus sample PFUs against GE content, as independently determined on the QuRapID under BSL4 testing using AR14 pre-qualification runs, reveals strong linear concordance between two Ebola virus strains diluted in FBS and the ArmoredRNA surrogate template (99.96% probability of single curve fit, Akaike's Informative Criteria test; individual replicates and 95% confidence bands shown).

Thus, we assembled a Peltier thermal cycling rig around the custom blood-compatible spectrophotometric emission/detection array used in our earlier experiments, modified through use of a heat removal module (HRM). This is a fluid cooled heatsink whereby the fluid, in this case at 35 °C, is held at a temperature intermediate of the PCR extension temperature and the freezing point (WO2010010361) to enable rapid –20 °C cooling. This relies on high levels of thermal isolation of the heatsink from the sample such that the Δ*T* of the Peltier can be relied upon to drive the reaction fluid to in excess of 50 °C below that of the HRM and hence make possible freezing in higher temperature environments. In these tests, we also used the Trombley+ assay and 10^5^ GE of our custom-manufactured AR14 EBOV Armored RNA® MS2 coliphage construct. Finally, given our results on RT-PCR enzyme tolerance for blood, we performed this test with the ABI Fast virus master mix, which is used in the EZ1 diagnostic assay. With the device achieving overall sample-to-answer in 70 minutes ahead of any additional RT-PCR thermal cycling timing optimization beyond the standard Taqman® cycling timings used in the EZ1 assay, these results reproduced the outcome of the manual study, identifying 8–10 freeze–thaw cycles as the optimal viral particle disruption range (Fig. 5a, S7A and B†). Moreover, the experiment confirmed that denaturation was not necessary for accessing viral nucleic acids for this form of viral particle disruption, even for the highly robust MS2 coliphage virions.

We therefore proceeded next to examine Trombley+ assay tolerance for blood by progressively increasing blood input from 0% to 10% v/v in reactions containing 6.6 × 10^8^ GE per reaction AR14 (Fig. 5b, S7C and D†). Thus, with the blood-free reactions returning a Ct of 15, an <128× loss of signal (Ct of 22) was observed at low blood concentrations; however, the trend was reversed to peak at 8% v/v blood with only a 4× signal loss (*i.e.* Ct 17). Crucially, this small signal loss only affected the assay lower limit of quantification (LLOQ; Fig. 5c), as 66, and 6 GE of AR14 were successfully discriminated from a no template, 8% v/v blood-containing reaction, but the two positive samples were indistinguishable between them. These results indicated that, although quantification at the lower end of the Trombley+ assay could be compromised in the presence of blood, detection could still be reliably achieved. On the basis of the 1.4 × 10^4^ PFU per ml whole blood detection limit for the laboratory-based Trombley assay,^9^ we reasoned that use of 5 microliters of whole blood in our method, *i.e.* 70 PFUs per reaction in a volume compatible with digital venipuncture, would match the performance of the laboratory method whilst remaining above the limit of quantification exhibited experimentally. Thus, the total reaction volume for our method was finalized at 62.5 μl containing a 5 μl blood sample (8% v/v). To determine whether accurate sampling at this scale could take in the place in the field we evaluated a range of IVD-grade, graduated plastic capillary samplers to observe a <3% standard deviation in 5 μl collection with the Pasteur pipette-like MicroSafe® capillaries (*n* = 20). This was conducted by operators wearing BSL4 personal protective equipment comparable to that used by frontline healthcare workers in triage centers.

Having achieved an assay chemistry, detection system and sample volume predicted to match the diagnostic reliability of the laboratory-based Trombley assay, we proceeded to construct a prototype point-of-care instrument (Fig. 4d). Importantly, this was designed for use in triage scenarios *e.g.* in emergency response primary care centers or in the vehicles of mobile outbreak response teams seeking to Quantitatively, Rapidly Identify (QuRapID) EBOV disease in symptomatic patients either off mains power or a car alternator power source. Initially designed as a lightweight, A4-foot print format, the final, 20 kg tabletop prototype sought to reduce the risk of unauthorized removal from triage centres and ensure robustness during transportation in *e.g.* off-road settings. In addition, this tabletop instrument featured 8 random access assay stations to allow for independent, parallel operation and a touch screen interface compatible with gloves. A SOP was also devised (ESI video online and Fig. S8†) in line with the simplicity necessary for minimally trained frontline staff focusing on patient care in a high stress environment whilst in full personal protective equipment. This included consumables design, automated buffer dispensing and secured thermal cycling that would minimize risk of environmental contamination, operator error or accidental sample release.

### Analytical sensitivity and specificity testing with live Ebola virus

With the outbreak ending by this time (November 2015) and the very limited number of new cases, the means for delivering evidence of clinical relevance with live EBOV were restricted to either stored samples or culture-derived EBOV tested in simulated patient samples. Previous reports indicated stored blood samples impacted efficient completion of PCR,^18^,^25^ which motivated us to seek samples stored in the absence of additives known to impact PCR. However, anecdotal evidence indicated substantial operator variability in sampling and storage procedures throughout the West African outbreak. Therefore, to minimize the impact of such variability inherent to stored samples *vs.* what should be a minimal, direct sample analysis process, we elected to proceed with BSL4 laboratory testing with a range of live, culture-derived Filoviruses. On the other hand, collection of fresh human blood by digital venipuncture was prohibited in BSL4 for health and safety reasons. As a result, fetal bovine serum (FBS) obtained without the use of anticoagulants or preservatives was used as a surrogate biological matrix to human blood. In doing so, we also acknowledged that direct volume substitution of blood with serum would double the final concentration of serum components in our reactions, doubling in parallel their PCR inhibitory effects.

Thus, the performance of the blood-compatible Trombley+ GP assay (Quasar 670/BHQ2) in ABI Fast virus master mix and our QuRapID prototype was evaluated against the Trombley/ABI7500 FAST gold standard SOP with the same mastermix, the FAM-labelled GP assay, and a variety of Filoviruses (Table S2†). After undertaking assay transfer to BSL4 and pre-qualification with our AR14 standards (Fig. 5d and S7E†), infectious, culture-propagated Filoviruses were serially diluted and tested at each resulting concentration on the QuRapID. Alternatively, the serially diluted viruses were extracted, purified and assayed according to the EZ1 assay SOP. The results indicated the Trombley+/QuRapID system could detect as little as a 1.4 × 10^3^ PFU per ml whole blood-equivalent of live EBOV Yambuka Ecran (Fig. 5d and S7F†), with a LLOQ of 1.4 × 10^4^ PFU per ml whole blood-equivalent. More importantly, however, performance across capsid surrogate viruses, EBOV Yambuka-Ecran and EBOV Makona Guinea was consistent in terms of the analyte that is quantified by RT-qPCR *i.e.* genome equivalents, rather than the number of viable virions (Fig. 5e). Crucially, no false positives were encountered across template-free controls or any of the genetically related viruses, confirming that the analytical specificity of the assay^7^,^9^ had not been compromised in the presence of FBS and fluorophore migration to far red spectra. Furthermore, these data were obtained through independent experiments carried out across seven days using multiple, separate virus types and batches. Nevertheless, and despite the propensity of PCR technologies for manual error, the results followed a log-linear relationship of virus genome concentration to Ct call, with 99.96% probability of single curve fit, at a linear regression *R*^2^ of 0.9967. These results indicated that assay performance and relative quantification could be pre-calibrated using MS2 Coliphage under BSL2 safety standards, with the inhibitory effect of FBS (and blood) not affecting assay linearity.

On this basis, to further investigate the extent to which the data generated by the two platforms were indeed comparable, we independently calibrated each dataset against standard curves generated using AR14, and calculated the unknown filovirus stock concentration in GE per ml. This analysis further indicated that the QuRapID platform returned data comparable to the gold standard lab-based Trombley method (Table 1). However, it also highlighted that where use of manual extraction/purification might be more sensitive to non-infectious viral genomes through analyte stabilisation, the QuRapID whole blood assay might be less so. Thus, the number of viral genomes to viable virions (log_10_ GE/log_10_ PFU ratio) for EBOV Yambuka-Ecran was higher than that of the younger EBOV Guinea Makona isolate irrespective of the platform used, but consistently lower on the QuRapID. These results were in line with separate reports^9^,^13^,^50^ which have suggested virus more extensively passaged *in vitro* might produce fewer infectious virions *vs.* newer isolates, thereby increasing the ratio of genomes to infectious particles in a biofluid.

**Table 1:** Comparative performance of manual *vs.* QuRapID-based quantification of live Ebola virus

Strain	Ebola virus amount			Genome/virion ratio (log GE/log PFU)	
	Live virions (log PFU per ml)	Genomes (log GE per ml)			
		ABI^*a*^	QuRapID^*b*^	ABI^*a*^	QuRapID^*b*^
*Zaire ebolavirus* Yambuka-Ecran (1976)	6.85	9.68	9.39	1.41	1.37
*Zaire ebolavirus* Guinea Makona (2014)	8.32	9.26	8.27	1.113	0.994

^*a*^Calibrated against quantified AR14 on the ABI7500 (ABI).

^*b*^Calibrated against quantified AR14 on the QuRapID (QR).

## Discussion

The socioeconomic value of molecular diagnostics in containing highly infectious disease has been convincingly demonstrated in the West and concurrent Central/East African EBOV outbreaks,^51^ and the South Korean Middle East Respiratory Syndrome Coronavirus (MERS-CoV) outbreak.^52^ Furthermore, historical data causally link international travel and mass attendance events with pandemic transmission.^53^ Therefore the capacity to deploy appropriate, reliable and cost-effective diagnostic capability is an international public health requirement. Yet, to date, there exists no true point of need solution suitable for mass screening that can support effective containment at international ports of entry or outbreak triage centers *etc.*, with minimal economic burden, transportation and international trade disruption. Indeed, we expect no such single system to prove of universal utility given transmission, symptomatology and carrier status may differ vastly depending on the biology of each pathogen and thus be best detectable through use of distinct analytical procedures. Rather, a flexible approach needs to be adopted, centered on responding rapidly, implementing appropriate technologies and proportionate procedures relevant to key factors to patient presentation and disease dissemination. Thus, whilst many highly infectious viral diseases may disseminate through asymptomatic carriers, EBOV transmission requires viraemia whose levels correlate with symptom intensity and indeed survival potential.^9^,^10^,^54^,^55^ As a result, many efforts have been made to detect pre-symptomatic levels of EBOV infection,^56^ with several anecdotal reports of various NAATs detecting pre-symptomatic individuals in the course of their evaluation during the West African outbreak. At present, however, the most reliable evidence thereto pertains to an 8-plex host microRNA biomarker signature detectable by NAAT in blood plasma.^57^ However, this has only a 50% pre-symptomatic, and a relatively low 86% infection status classification potential. Therefore, reliable containment of future EBOV outbreaks will pivot on effective detection and screening of symptomatic individuals. Crucially, such EBOV positive patients typically present with viraemia levels well in excess of 10^5^ GE per ml of whole blood, *i.e.* some 2–4 orders of magnitude above of the LLOD of the Trombley and other RT-qPCR assays, as demonstrated in multiple studies during the West African^4^,^55^,^58^,^59^ as well as historical outbreaks.^3^–^5^,^60^

Of the diagnostic solutions proposed for EBOV and other infectious diseases, the most advanced, minimal end-user input technologies include antibody lateral flow tests^61^ and semi-automated NAATs.^10^,^17^ The latter, however, remain the most reliable and robust methods for diagnosing EBOV in viraemic patients,^9^,^10^,^54^ with antibody systems suited to exclusion screening in cadavers or patients exhibiting intense symptoms.^62^ Yet both NAATs used for diagnostic purposes in West Africa^7^,^63^ required mobile laboratory deployments.^2^ Thus, the benchtop BioMérieux BioFire® FilmArray and Cepheid GeneXpert® NAATs received WHO and/or FDA EUA during the West African outbreak. Both instruments require basic laboratory infrastructure, are semi-quantitative, multiplexing-compatible and up to eight times faster than the manual Trombley method. Whilst BioFire® offers FDA-approved, differential diagnosis at a cost of ∼US$150 per test, GeneXpert® assays for single agents at ∼US$20 and offers medium throughput through scalable devices. In both cases, however, sample processing still requires flexible-film isolator containment, technical expertise pertaining to the manual steps involved, and know-how related to data interpretation. Nonetheless, together, these solutions allow for index case causal agent identification and provide some capacity for screening, albeit still in a laboratory setting and at considerable cost largely due to microfluidics and/or pumps which mechanise as many manual procedures as possible. Thus, overall, BioFire® tests are comparably priced to laboratory-based Trombley assays, whereas the GeneXpert® EBOV test remains up to twice more expensive than the annual *per capita* healthcare spend in West Africa, despite charitable development funding and ex-works pricing as negotiated by FIND Diagnostics on behalf of the WHO, substantially reimbursed by the Gates Foundation. Importantly, the recently announced, portable GeneXpert® Omni offers single sample analysis capacity albeit with no improvement in sample-to-data turnaround (2 hours), questioning its mass screening utility. Moreover, both systems are ‘closed access’, meaning public health authorities cannot independently respond to emerging threats by rapidly transitioning their in-house developed assays to such disseminated use platforms: tellingly, the Trombley assay received FDA EUA days after the WHO PHEIC declaration, whereas the Cepheid platform required several months of additional development. Elsewhere, emerging alternative NAAT solutions focus on the energy efficiency of isothermal amplification technologies^64^ as well as the agnostic, *de novo* analytical power of memory stick-sized sequencing systems.^65^,^66^ Whilst many of these have been successfully evaluated in resource-limited settings,^64^,^65^ these solutions still require extensive and costly laboratory infrastructure for sample pre-processing^64^–^66^ even onto genome extraction,^65^,^66^ are non-quantitative,^64^ closed access,^64^,^66^ or require high bandwidth data transfer/computational power access^65^,^66^ to realize their potential.

In this work, we have demonstrated that controlled, low temperature freeze–thawing of viruses as structurally diverse as MS2 coliphage (Armored RNA®) and EBOV can release virion genomes within a complex biofluid matrix such as serum or fresh blood. Furthermore, we have evidenced that simple substitution of commonly used green DNA stains and probe dyes with red/far red fluorophores spectrally compatible with blood eliminates fluorogenic inhibition in RT-qPCR. Unlike previous reports involving various formats of stabilized blood,^18^,^19^,^25^ in our hands, use of freshly obtained blood, *e.g.* by digital venipuncture, did not appear to extensively or entirely inhibit PCR. More importantly, combination of controlled freeze/thawing with far red fluorophores and simple RT-qPCR enzyme formulations was found to be reliable enough to adequately and co-linearly detect specific genetic targets across at least two classes of viruses, assayed on separate days. To our knowledge this is the first use of pre-calibration standards for RT-qPCR-mediated sample quantification, directly in whole blood, across a wide dynamic range, with minimal impact on sensitivity and no impact on diagnostic specificity. Thus, the 1.4 × 10^4^ PFU per ml of whole blood equivalent detection limit for the Trombley+ assay on the QuRapID platform is approximately one order of magnitude higher than the detection limit of the gold standard laboratory-based Trombley/ABI7500 test. At first glance this might appear too great a difference between a novel and a gold standard platform. However, the Trombley+/QuRapID LLOD is comparable to the independently reported LLOQ of the Trombley/ABI7500 gold standard at 1.2 × 10^4^ PFU per ml.^9^ Nonetheless, this metric does not relate to viral genomes, the analyte detected by both assays. Rather, performance qualification is expressed relative to the concentration of viable, infectious viral particles, which are impossible to quantify by RT-qPCR. However, both our data and the latest independent Trombley assay performance report,^9^ present GE/PFU ratios: this makes direct comparisons acceptable across the studies, laboratories and platforms. Thus, in the context of the 1 × 10^5^ GE per ml or greater viraemia levels exhibited by symptomatic EBOV patients,^3^–^5^,^55^,^58^–^60^ our results evidence clinical utility for our proposed approach to meet demand during future EBOV outbreaks, subject to completion of the necessary EUA qualification studies. Moreover, as EBOV caches in the cerebrospinal fluid of survivors and may lead to meningoenchephalitis,^67^ we have also briefly examined whether our method is compatible with this biofluid. Thus, we observed no effect from blood contamination on Trombley+/QuRapID Ct calls after spiking AR14 at 1 × 10^8^ GE into reactions containing 10% v/v human cerebrospinal fluid (Fig. S9†). The approach may therefore offer utility beyond symptomatic EBOV diagnosis, not only to other infectious viral disease with viraemia levels relevant to the detection limits of our methodology, but also to the rapid and economic detection of the causes of meningitis where time-to-diagnosis through 3 day-long, classical clinical microbiology might be disadvantageous. Most importantly, however, unlike many other approaches, the performance we report with this novel platform is achieved with 700× less blood volume than the standard 3.5 ml needed for the gold standard Trombley assay. Studies are therefore underway to explore whether large volume PCR^68^ accommodating higher amounts of patient sample at the point of care might meet and even exceed the sensitivity of the EZ1 SOP.

Beyond sensitivity and specificity, in line with the ASSURED criteria,^16^ key to disseminated use of novel technologies, and especially in resource limited settings, is test affordability. In its present, diagnostically relevant, 62.5 μl reaction format, disposables for the Trombley+ assay on the QuRapID cost US$12.50 per test before scale up or subsidy, an amount relevant to developing nations and competitive to emerging electricity-free,^69^ or disposable isothermal NAAT that rely on complex fabrication methods.^70^ Thus, in the context of powered point-of-need solutions, the 2 minute, 10 cycle freeze/thaw process implemented in our approach adds only an additional 24 watts of energy requirement per test. This can be nonetheless additionally managed through smart thermal load sharing between active stations operating *ad hoc*, thereby reducing overall energy requirements during screening use. Moreover, the overhead is effectively compensated by the lack of any motorized, expensive consumables or instrument parts that could otherwise compromise instrument robustness. To extend this principle to overall process user-friendliness, we have additionally minimized to the greatest extent possible the manual steps involved in conducting RT-qPCR (ESI video online†) without recourse to laboratory infrastructure or introduction of manufacturing complexity. Thus, the standard operating procedure we devised involves a disposable consumable, pre-loaded with lyophilized reagents and an automated, pre-calibrated 57.5 μl solvent dispenser to eliminate the need for refrigeration. Although the ABI Fast RT-qPCR mastermix we tested against live EBOV is not in itself amenable to lyophilisation, refrigeration-free RT-qPCR mastermix production is possible^71^ and commercially available for research and diagnostic purposes. To this end, we have screened and identified a proprietary, lyophilisation- and blood-compatible formulation, successfully dried and tested it as part of this work; this formulation is presently under development to match the wet assay performance characteristics reported herein.

An essential requirement in any diagnostic technology, particularly for hazard group 4 pathogens, however, is safety: this takes the form of both operator safety and diagnostic reliability. Although the use of a flexible film isolator for the QuRapID instrument would be subject to local health and safety requirements in an ‘orange’ or ‘red’ triage zone, IVD-marked, low operator risk, sheathed lancets for digital venipuncture are readily and cheaply available over the counter *e.g.* for use with point of care glucometers. In our hands, these can be safely handled in full personal protective equipment suitable for hazard group 4 pathogens. Use of plastic capillaries, such as the low error rate 5 μl Microsafe® trialed herein, can also eliminate the risk of injury or instrument contamination beyond the puck consumable. Similarly, the optical lid is additionally secured in place on the QuRapID during thermal cycling by the weight of the top-down detector, which is itself magnetically held in place. Thus, after process completion, all consumables can be disposed of as hazardous clinical waste, and the instrument can be wiped down or indeed fumigated. Crucially, no instrument, electronics or optics damage or failure was observed after fumigation and exit of our prototype QuRapID platform from BSL4 containment after testing with live EBOV. Therefore, the platform retains the mobility and repeated utility requirement pertinent to rapid response in outbreak scenarios.

On the other hand, experimental evidence is required supporting accurate detection of multiplexed RT-qPCR to support safe calling of process and diagnostic success during use of this technology. Unfortunately, in our hands, the singleplex Trombley assays and the RP control assay exhibited competitive inhibition at the lower end of analyte concentration. Reasoning that altering even primer and probe concentrations would require re-qualification of assay specificity, we decided instead to confirm the utility of our approach against EBOV genome detection in singleplex mode. Nevertheless, the resulting fluorimetric data were used to examine state of the art mathematical deconvolution approaches used in other optical platforms operating in the same spectral ranges. This analysis indicated that separation of commercially available probe fluorophores in the 600–750 nm range is adequately assayed spectrophotometrically, and that the signal to noise ratio of a fluorophore might direct its use in a multiplexed fashion. Thus, an example dye trio consisting of CAL635, Quasar 670 and Quasar 705 could enable migration of an established triplex assay to the far red, blood-compatible spectrum, but the signal to noise ratio of Quasar 705 in the context of blood would make this fluorophore suitable only for the detection of a high copy number control. Encouragingly, the spectral data obtained throughout these studies with Quasar 670-labelled probes are in line with our predictive modelling efforts regarding peak ratiometry. Thus, fluorigenic data normalization clearly exhibits adequate assay window in reliably detecting emission peak changes as no deviation in ratiometric profiles was detected for Quasar 670 relative to other dye emission peaks. Efforts are thus underway to implement automated Ct calling in multiplex mode as well as viral genome quantification through reagent pre-calibration based on our observations across AR14 and the two distinct EBOV strains we tested. Overall, whether substitution of probe-based detection chemistry with DNA stains and high-resolution melt will enable further cost reductions in the future will largely depend on the relative diagnostic reliability, computational, and energy requirements of the two approaches when used in the presence of blood *vs.* already available, highly reliable probe assays.

In this context, in response to the 2015 MERS-CoV South Korean outbreak, time-to-transfer for an established probe chemistry assay onto the QuRapID platform and far red, blood-compatible chemistries was calculated at six to eight weeks, with rate limiting steps being (a) surrogate BSL2 template production, and (b) reagent synthesis. Thus, stockpiling notwithstanding, we estimate that BSL4 laboratories with robust reagent supply chains could independently deliver mass screening, mass production-ready assays within a fortnight. The limited impact of established assay migration to blood-compatible chemistries observed herein, coupled to readily available, high confidence computational assay design capability and parallel screening capacity even suggests utility against pathogen genomic drift, to enable rapid response for newly identified pathogens.

## Experimental

### Primers, probes and fluorescent dyes

We focused on the Trombley primer and probe sets for the EBOV GP and NP genes,^7^,^9^,^10^ considering the human RP assay as a control,^8^ and an MS2 *Escherichia coli* phage-specific assay set^72^,^73^ for fluorescent probe- or intercalator-based blood tolerance studies (SYBR Green I; Thermo Fischer Scientific, Warrington, UK). Primers (Table S3†) were synthesized by Integrated DNA Technologies (Leuven, Belgium). Probes were purchased from Thermo Fisher Scientific (FAM), IDT-DNA (FAM, Cy5; Leuven, Belgium), and LGC BioSearch Technologies Ltd. (Quasar; Belfast, UK). Computational PCR performance analysis was carried out using VisualOMP™ v.6.3 (DNA Software, Ann Arbor, MI, USA). Fluorophores were purchased from Thermo Fisher Scientific (instrument calibration, 1,2-diamino-4,5-dimethoxybenzene (DDB), and SYTO®), Fluorogenics Ltd. (Viridian Red), and LGC BioSearch Ltd. (ATTO and CAL635 dyes).

### RT-qPCR blood tolerance experiments

To determine the tolerance of commercially available RT and PCR enzymes to blood, one-step reaction master mixes were assembled on wet ice using GoTaq® G2 Hot Start polymerase (Promega; Southampton, UK) in GoTaq® G2 Hot Start Colorless Master Mix (Promega) supplemented with 5 units of GoScript® Moloney Murine Leukemia Virus reverse transcriptase (Promega), primers and probes/dyes at concentrations as previously published.^6^,^27^ Duplicate aliquots were dispensed, and supplemented with blood and nuclease-free water (Promega) to achieve the final blood concentrations indicated. Template was added last in each aliquot to achieve a 50 μl final reaction volume ahead of commencing thermal cycling on an Illumina Eco™ running Eco™ Software v.4.0.7.0 (Illumina Inc., San Diego, CA, USA).

### Surrogate virus standards and biological matrices

To enable accelerated development under biosafety level 2 (BSL2) we used a Hepatitis C Virus^74^ (HCV) Armored RNA Quant® (Asuragen, Inc., Austin, TX, USA; manual phase disruption studies) as an MS2 coliphage virion model and three EBOV surrogates (Table S2†): a custom Armored RNA Quant® (AR14; 10 (ref. 12) GE per ml stock concentration quantified against synthetic RNA templates by RT-qPCR) and two, in-house produced, pseudotyped lentiviruses. These surrogates featured RNA genomes engineered to contain the concatenated target sites for the Trombley GP and NP assays as encoded in the 1976 Yambuka-Ecran EBOV (PV76) or 2014 Guinea Makona (PV14; AR14).

To generate these recombinant lentiviruses, assay targets flanked by *Bam*HI and *Not*I (Geneart plasmids, Thermo Fisher Scientific) were sub-cloned^23^ into the transfer plasmid pDUAL-eGFP (a gift from David Escors, University College London). Lentiviruses were assembled in HEK293T cells grown in 10% v/v fetal bovine serum (FBS)-supplemented Dulbecco's Modified Eagle Medium (DMEM; Thermo Fisher Scientific), 5% v/v CO_2_, 37 °C, using a second-generation method involving 1 mg per ml polyethylenimine (Sigma Aldrich, Dorset, UK) co-transfection with pMD2.G (a gift from Didier Trono; Addgene plasmid #12259) carrying the envelope protein for vesicular stomatitis virus, and p8.91 encoding the lentiviral core genes,^75^ at a plasmid ratio of 1 : 1 : 1.5, respectively. Supernatants were replaced with 6 mL fresh media at 16 hours and 0.45 μm filter-harvested at 40 hours for 4 °C storage. Cells were fed with fresh media (5 ml) and Lentivirus was filter-harvested again at 64 hours, pooled, concentrated (4000 × *g*, 4 °C, 16 hours), re-suspended in 1.0 mL DMEM, re-centrifuged through a 5 ml, 20% w/v sucrose cushion and re-suspended in 1.0 ml DMEM for –80 °C storage. Yields (viral particle per ml (vp per ml) or genome equivalent per ml (GE per ml)) were derived using a Nanosight NS300 (Malvern Instruments, Malvern, UK) after 1 : 10 dilution in 10% v/v FBS-supplemented DMEM or interpolation against standard curves of AR14 RNA extracts by Trombley assay for the PV76 and PV14 constructs, respectively. RNA extracts were obtained using the QIAamp Viral RNA Mini Kit (Qiagen, Manchester, UK), according to the manufacturer's instructions, assuming 100% extraction efficiency.

Fresh human blood was aseptically obtained by digital venupucture of healthy adult male volunteers (Vitrex® Sterilance® LiteII lancets, VWR, Lutterworth, UK), as approved under the University of Westminster Research Ethics Committee (application no. VRE1415-0171, 7th Nov. 2014). Informed consent was obtained for any experimentation with human subjects. Cerebrospinal fluid (CSF) was kindly provided by Prof. T. Solomon (University of Liverpool). Patient samples were simulated under BSL4 using FBS (Thermo Fisher Scientific).

### Trombley assay transfer

Plate-based RT-qPCR assays were carried out on an ABI7500 (Thermo Fisher Scientific) in accordance with the manufacturer's SOP specified for the EZ1 assay. Briefly, after extraction/purification using the QIAamp Viral RNA Mini Kit into 70 μl eluents, RNA was serially diluted using nuclease-free water (Qiagen, Manchester, UK). Trombley assay one-step RT-qPCR^7^ was carried out on 5 μl samples in ABI Fast virus master mix (Thermo Fisher Scientific) at 20 μl final reaction volumes. Factional threshold cycle (Ct) values were obtained using the 7500 ABI software (v.2.3), implementing onset of amplification Ct calls. Performance metrics were derived on technical triplicates using electronic pipettes: data were represented as means ± 95% confidence bands (LDR testing) or triplicate average ± standard deviation, unless otherwise specified.

### QuRapID instrumentation

Pre-prototype work on phase transition was carried out using manual acetone/dry ice bath to 105 °C dry block cycling of standard 0.2 ml PCR tubes (Thermo Fisher Scientific). Sample temperature was measured in separate tubes loaded with equal volume mock samples and an alcohol thermometer to monitor ramping and hold rates. Instrument and software development of the novel QuRapID (Fig. 4d) thermal cycler (WO/2011/157989) involved a Raspberry Pi (Raspberry Pi Foundation, Cambridge, UK)-controlled, single-well prototype (Fig. 4c) with custom-fabricated thermal control printed circuit boards, to evaluate the complete Luxeon Rebel range of light emitting diodes (Lumileds, Amsterdam, Holland), filtered white light sources and laser diodes (Osram, Regensburg, Germany). Performance testing was carried out on two separate 8-well instruments in three separate laboratories (University of Westminster, BioGene Ltd. and Public Health England). Fluorophore and probe dye/quencher compatibility with blood was assessed in duplicate by adding 4–5μl of blood directly into a reaction master mix (50 μl final volume). Results were expressed as the absolute fluorescence, and summarized as the difference in fluorescence (positive or negative) in the presence and absence of blood after background-subtraction. Emission peaks were defined as the wavelength with the highest emission intensity for each dye, with peak emission intensity defined as the average intensity at ±1 nm from the specific wavelength (11 values total). Signal to noise ratios were calculated against the blood sample readings within the same wavelength region as defined by the dye measurements in the presence of blood. Threshold values were calculated as 1.5 standard deviations above the emission peak average intensity in the dye-free, blood background samples. Data processing was carried out on Microsoft Excel (v.15; Microsoft, Reading, UK) and graphical representation was performed on Microsoft Excel or GraphPad Prizm v.7.0a (San Diego, CA, USA). The end-user-directed SOP (video online) based on a 62.5 μl final reaction volume and 5 μl fresh blood samples (8% v/v final blood concentration in the reaction) is summarized in Fig. S7.†

### Biosafety level 4 (BSL4) sensitivity and specificity testing

The infectious viruses evaluated in this study (Table S2†) were specific stocks produced at the Porton Down BSL4 laboratory of Public Health England. Live viruses were quantified by titration to obtain 50% tissue culture infectious dose (TCID_50_) in VeroE6 cells (European Collection of Cell Cultures, UK) and titers were converted to PFU per ml by multiplying the TCID_50_/ml values by 0.7 assuming Poisson distribution.^11^

All live virus manipulations and QuRapID analyses were performed under BSL4 across 7 separate days including instrument pre-calibration runs on AR14. Live viruses were serially diluted 10-fold in FBS or minimal essential medium plus GlutaMax™-I (MEM; Thermo Fisher Scientific) and used as templates for the QuRapID-migrated Trombley GP assay (Trombley+ assay). Reactions comprised of 57.5 μl RT-qPCR master-mix (TaqMan® Fast Virus 1-Step Master Mix (Thermo Fisher Scientific), primers, and probes (Table S3†)), to which 5 μl of FBS-diluted virus or 4 μl FBS and 1 μl of MEM-diluted virus were directly added. Assay performance was expressed as Ct call against titration-derived infectious virus concentration in PFU per ml or genome content in GE per ml based on AR14 pre-calibration.

Independent samples across the two virus dilution series (140 μl per dilution) were chemically inactivated in accordance with PHE's hazard group 4 inactivation methods. Sample tubes were disinfected with detergent in accordance with PHE's BSL4 disinfection policy for safe working of material at BSL2. RNA was extracted using the QIAamp Viral RNA Mini Kit (Qiagen) and quantified against RNA extract standard curves from AR14 using an Applied Biosystems 7500 Fast (Thermo Fisher Scientific). Primer and probe sets specific to Filoviruses other than EBOV were used in specificity tests (Table S3†). Stock virion concentration in GE per ml was quantified as the titration range average, as calculated per analytical platform.

### Statistical methods

Linear regression analyses to define assay LDR were conducted on GraphPad Prizm on the means of technical triplicates, with 95% confidence bands graphically represented across the titration range tested. The probability of single curve fit for independent assays or assay templates was determined by Akaike's Informative Criteria Test conducted on blinded sample data using GraphPad Prizm. Diagnostic parameters were determined in accordance to Armbruster and Pry.^76^

## Authors contributions

S. A. M. conceived this research, selected with D. A. N. and D. E. the assays to be used herein and established with the assistance of D. A. N. and K. S. R. R. technology specification, standard operating procedure requirements and minimum acceptable performance criteria for system progression. S. A. M., D. E. and E. W. planned and oversaw execution of all BSL2 work presented herein, with K. S. R. R. overseeing all BSL4 work and M. L. consulting on EUA-grade assay development requirements. K. S., D. E., K. S. R. R. and S. A. M. designed the recombinant virion assay target sites. S. A. M., D. E. and K. S. evaluated manually, computationally and biochemically the impact of assay template genetic drift on assay performance. K. S. undertook with the assistance of L. U. all BSL2 assay transfer, modification and validation work. K. S., E. B. and E. W. constructed the recombinant HIV pseudotyped virions (BSL2 standards). C. C. and D. L. performed all RT-qPCR in blood inhibition and blood-compatible lyophilized assay development studies under the guidance of M. L., S. A. M., D. E. and N. N. S. A. M. and D. E. proposed the use of red dyes for blood-compatible RT-qPCR. D. E., A. T. and K. S. established the optimal conditions for blood-compatible single- and multi-plexed RT-qPCR, with S. A. M. conducting multiplexing modelling. N. N. oversaw implementation by D. E. and A. T., of instrument/consumable engineering, software and standard operating procedure specifications set by S. A. M. as advised for in-country requirements by K. R. and D. A. N. L. E., J. B., C. B. and K. S. R. R. performed all BSL4 procedures described herein. S. A. M., K. S., K. S. R. R., D. E. and A. T. prepared this manuscript with equal support from all contributing authors.

## Conflicts of interest

The authors declare no competing financial interests.

## Supplementary Material

Supplementary information

Supplementary movie
