# Supplementary material for: Field-deployable, quantitative, rapid identification of active Ebola virus infection in unprocessed blood
Source: Chem Sci. 2017 Sep 25;8(11):7780–97. doi: 10.1039/c7sc03281a (PMC5694917; doi:10.1039/c7sc03281a)
Supplement: Supplementary file 1 [file SC-008-C7SC03281A-s001.pdf]

Electronic Supplementary Material (ESI) for Chemical Science.
This journal is © The Royal Society of Chemistry 2017

Field-deployable, Quantitative, Rapid Identification of Active Ebola Virus Infection in Unprocessed Blood.

Kavit Shah MSc,[1, 2] Emma Bentley BSc,[3] Adam Tyler PhD,[4] Kevin S. Richards PhD,[5] Edward Wright PhD,[3] Linda Easterbrook MSc,[5] Diane Lee MSc,[6] Claire Cleaver MSc,[6] Louise Usher MSc,[1] Jane E. Burton PhD,[5] James K. Pitman BSc,[5] Christine B. Bruce PhD,[5] David Edge PhD,[4] Martin Lee PhD,[6] Nelson Nazareth MBA,[4] David A. Norwood PhD,[6] and, Sterghios A. Moschos PhD.*[1, 3, 8]

1. Westminster Genomic Services, Department of Biomedical Sciences, Faculty of Science and Technology, University of Westminster, 115 New Cavendish Str. London W1W 6UW, UK.

2. BGResearch Ltd., 6 The Business Centre, Harvard Way, Harvard Industrial Estate, Kimbolton, Huntingdon PE28 0NJ, UK.

3. Department of Biomedical Sciences, Faculty of Science and Technology, University of Westminster, 115 New Cavendish Str. London W1W 6UW, UK.

4. BioGene Ltd., 8 The Business Centre, Harvard Way, Harvard Industrial Estate, Kimbolton, Huntingdon PE28 0NJ, UK.

5. Public Health England, National Infection Service, High Containment Microbiology Department, Porton Down, Salisbury, Wiltshire, SP4 0JG, UK.

6. Fluorogenics LIMITED, Building 227, Tetricus Science Park, Dstl Porton Down, Salisbury, Wiltshire, SP4 0JQ, UK.

7. Diagnostic Systems Division, and Virology Division, United States Army Medical Research Institute of Infectious Diseases, Fort Detrick, MD 21701-5011, USA.

8. Department of Applied Sciences, Faculty of Health and Life Sciences, Northumbria University, C4.03 Ellison Building, Ellison Place, Newcastle Upon Tyne, Tyne and Wear, NE1 8ST, UK.

Electronic Supplementary Information

Standards and surrogate template construction.

MS2 coliphage is a 20 nm, non-enveloped, icosahedral virion with a positive RNA genome commercially available as Armored RNA®. It is considered a highly resilient particle that is used as a process/spike-in control for laboratory-based RT-qPCR involving complex RNA extraction and purification steps, e.g. in the Cepheid Xpert® RT-qPCR tests for human immunodeficiency virus (HIV). Filoviruses, however, are distinct to MS2 as their 80 nm x 14 μm, string-like particle consists of a negative RNA strand packaged into a ribonucleoprotein complex surrounded by a lipid bilayer studded with glyocoprotein. More importantly, filoviruses are considered very unstable and easily fragmentable.[22] Given the higher stability of Armored RNA®, we reasoned that using recombinant MS2 as a model would set a more stringent performance bar to our work. We therefore procured commercially a custom Armored RNA® standard (AR14) engineered to contain the concatenated amplicon sequences corresponding to the Trombley GP and NP assay targets, as encoded in the reference genome of the West African EBOV outbreak strain, EBOV Guinea-Makona (ESI Fig. 1). As a fallback, however, we also produced in-house recombinant, pseudotyped lentivirus (PV) based on HIV using our established triple plasmid approach.[23] Unlike MS2 coliphage or indeed EBOV, the HIV virion is a ~120 nm particle consisting of a double stranded RNA genome packaged in a capsid, which is itself encapsulated in a glycoprotein-studded, lipid-envelope. The AR14 standard was supplied at 10^{12} GE/ml stock concentration as determined by supplier RT-qPCR using synthetic RNA standard curves, whereas PV constructs were quantified using nanoparticle tracking analysis after dilution in serum-free phosphate buffer saline (PBS; ESI Fig. 1B).

Impact of EBOV genetic drift on the Trombley diagnostic assay.

We also took the opportunity to explore the impact of EBOV genetic drift on molecular diagnosis through our in-house produced PV standards. Thus, epidemiological deep sequencing data from West African EBOV isolates[24] indicated single point mutations in the 2014 outbreak EBOV Guinea Makona genome as compared to the 1976 EBOV Yambuka-Ecran reference that had been used to engineer the Trombley assays (ESI Fig. 1). These differences were suggested[24] to have minimal impact on diagnostic assay performance but remained untested. In line with these reports, nearest neighbor primer/probe hybridization computational modelling in our hands also indicated minimal changes for the affected primer and probe hybridization free energies (ESI Table 1). To confirm this experimentally we therefore manufactured two PV constructs: one featuring the Trombley assay target insert corresponding to EBOV Guinea-Makona (PV14), and one featuring the polymorphisms found in EBOV Yambuka-Ecran (PV76; ESI Table 2; ESI Fig. 1B). After extracting RNA from PV construct cell culture supernatants using the QIAamp Viral RNA Mini Kit (Qiagen, Manchester, UK), we performed RT-qPCR assays on serial RNA dilutions to evidence minimal differences on the GP assay reaction efficiency between the 1976 reference genome (104%; %CV <1.07%) and the 2014 outbreak genome (102%; %CV <3.58). Differences in assay efficiency when using the AR14 template (98.0%) or synthetic RNA as reported elsewhere (93.8%),[9] were attributed to the distinct sequences flanking the amplification template in each construct, the template virion production methodology, and the infectious virion:GE ratio effect.[9,12,13] Thus, our assay template inserts had been cloned within the 9.2 KB dsRNA PV14 genome extracted from eukaryotic cell culture supernatant or the 3.6 KB single stranded RNA AR14 genome produced in bacterial broth culture supernatants, as opposed to the relatively high purity, 55bp synthetic RNA template for the GP assay used by others.[7] However, given the limited variance observed between the two PV constructs, these data reinforced the premise that EBOV genomic drift effected limited impact on diagnostic assay reliability.

Modelling far red dye multiplexing in the presence of blood.

Mathematical linear[42] and non-linear[43] unmixing are spectrophotometric approaches well-established in confocal microscopy and flow cytometry that allow for highly reliable[44] deconvolution of individual fluorescent dye contributions to a composite fluorescence trace. In its simplest form,[42] a composite spectrum is the sum of individual dye contributions at a given wavelength, each contribution being the product of dye concentration multiplied by its reference (i.e. normalized) emission at the specific wavelength. Consequently, the specific contributions of each dye can then be calculated via linear least squares fitting, assuming dye-dye interaction that would alter their spectra. To model whether this might be possible to implement in qPCR and RT-qPCR through spectrophotometric signal acquisition, we first compared the emission spectrum overlap for a selection of far-red dyes as measured in blood-containing solutions (ESI Fig. 5A). In conjunction with the established peak signal to noise ratios for these dyes within a blood matrix, this comparison indicated that adequate emission peak separation might facilitate spectrophotometric signal deconvolution, e.g. when combining Quasar 670 with Quasar 705 (duplex) and even CAL635 (triplex).

Thus, we proceeded to test whether multiplexing the GP, NP and RP assays was readily possible. However, in the absence of any additional reaction optimization, we observed competitive inhibition that resulted to loss of sensitivity. Reasoning that alteration of the Trombley assay specifications to facilitate multiplexing (primer and probe concentrations, let alone oligonucleotide sequences) would require revalidation of analytical specificity, we proceeded to computationally determine the key parameters that would facilitate probe-based RT-qPCR multiplexing by spectrophotometry in the presence of blood.

PCR is a kinetic process that requires accurate signal deconvolution at each cycle to efficiently detect the onset of exponential template amplification, and thereby derive an estimate of template quantity. Moreover, unlike the continuous nature of fluorogenic data in microscopy, in qPCR, the fluorogenic signal at the emission peak of each dye follows a discontinuous sigmoidal curve (e.g. Fig. 2), with the linear segment characterized by a 2-fold fluorogenic signal increase typically spanning 6 PCR cycles before a plateau is reached. This corresponds to an ~3 $\log_{10}$ range of fluorogenic signal increase from background. Interestingly, kinetic spectral ratiometry[45] has been successfully used to improve deconvolution accuracy. As a result, the composite spectra of dye mixtures at defined concentration ratios are plotted and spectral waveform cross-correlation analysis is used to eliminate intensity artefacts; importantly, the method also accommodates linear and non-linear relationships. The resulting plots are then used as ratiometric references to define individual dye contributions in unknown spectra, expressed as dye::dye ratios. This is similar to the use of endogenous control genes in qPCR as reference points for determining relative gene expression levels (the $2^{-\Delta\Delta Ct}$ approach).[46] An added complexity in our application however, was the background emission spectrum of blood. Background (tissue) fluorescence is indeed an issue in fluorescence microscopy, however to the best of our knowledge, this has not been factored in kinetic spectral ratiometry. We therefore decided to model the utility of such computational approaches in probe-based qPCR and RT-qPCR spectral de-multiplexing, in the context of a blood-containing reaction.

Using blood spectra as a background threshold, and the experimentally derived, fluorogenic emission data of the dyes in the presence of blood as maximum readings, we constructed composite emission spectrum reference tables for combinations of 2 and 3 dyes, spanning a range of 6 individual dye concentrations. These concentration ranges corresponded to the theoretical 2-fold fluorescence increase per PCR cycle across the 6 cycles typically involved in the exponential phase of 100% efficient qPCR. Focusing on the emission peak of each dye, we then compared the composite signal level to the background blood emission intensity. Importantly, we set 2.5 standard deviations over the local (+/- 1nm) emission in the blood spectrum as a background threshold, given this safely exceeded the local background

maximae for all dye peaks. This analysis indicated that for the CAL635-Quasar 670 combination, amplification would be clearly detected for either target, as the composite emission signal at either 635 nm or 670 nm would remain below the blood background only if neither target amplified (ESI Fig. 5B, C). Use of the Quasar 705 dye, however, would restrict the composite emission signal below background at 705 nm irrespective of the alternative dye used (ESI Fig. 5D, E). Moreover, Quasar 705 would also impact upon detection at 635 nm or 670 nm for CAL635 (e.g. ESI Fig. 4F) or Quasar 670, respectively. On the other hand, in triplex format, the effect of Quasar 705 would be limited only to the 705 nm peak (ESI Fig. 4H-J). These results suggested that Quasar 705 would be useful only for the detection of high copy number targets where a fluorogenic maximum would be guaranteed, such as endogenous control genes or spike in controls. Thus, a pre-requisite for using Quasar 705 effectively in combination with CAL635 and Quasar 670 would be ensuring early amplification of the Quasar 705 target to avoid any loss of sensitivity for the second or third fluorescent dyes.

Testing the compatibility of far red fluorescent probes with fresh blood.

For these experiments, a dual core fiber set up was used, operating as a reflection probe and 5mW fiber coupled laser diode (ProPhotonix, Salem, NH, USA) at 635nm as the excitation source. The laser diode was coupled into an 0.22NA 200 μm Y fiber. The excitation light was filtered with an in line 650nm shortpass filter (Edmund optics, York, UK). The collection fiber was placed 0.7mm from the clear lid of the vessel and the spectrophotometer leg had a 650nm longpass filter to reject the laser line. The readings were taken on a MAYA LSL (Ocean Optics, Edinburgh, UK) with a 100ms integration time.

All probes exhibited substantial emission peak signal increases in the presence of 8% v/v fresh human blood, with the exception of the Quasar 670/BHQ3 combination (ESI Fig. 4H). Thus, whilst use of BHQ2 increased peak intensity by 66% (ESI Fig. 4G), use of BHQ3 reduced it by 54.5%. Although at first glance this was attributed to quencher strength and probe stability, this may not be the case. Thus, whilst BHQ3 is a more powerful quencher, it is considered less stable than BHQ2. Accordingly, substitution of BHQ2 with BHQ3 reduced the peak intensities of both Quasar 670 and Quasar 705 in the absence of blood. The effect, however, only applied to Quasar 705 in the presence of blood, which would suggest blood caused the loss of BHQ3 in the Quasar 670 probe. Yet loss of BHQ3 quenching would be expected to lead to fluorogenic signal increase rather than the observed decrease. Alternatively, as the same oligonucleotide was used across these experiments, these findings might be indicative of altered membrane permeability and/or protein::probe interactions with cellular, extracellular and exosomal components in the blood matrix, on account of the physicochemical properties of individual quenchers in the context of specific fluorophores. This is a frequently overlooked issue, even though it has previously been shown to dramatically alter observations in cell[34] and animal[35] assays.

Freezing-denaturation release of virion genomes for RT-PCR: proof of principle

No thermal cycler, let alone a real-time instrument, was available at the time capable of cooling to sub-0°C to freeze an RT-PCR reaction mix. This restricted our studies to manual thermal cycling between -10°C and 95°C using an acetone/dry ice bath and dry heat block set to 105°C. To take advantage of the lead time to the production of our EBOV surrogate standards, we also used an alternative Armored RNA® QUANT construct and its diagnostic grade end-point RT-PCR primer set, as these materials were immediately available off-the-shelf. However, as this assay had not been evaluated for utility in DNA stain-based RT-qPCR, and to avoid delays thereto, we explored whether limiting virus copy number (500 copies or less) and the extent of freezing/denaturing (0-15 cycles) contributed to successful template detection. Thus, if viral RNA release occurred due to osmotic lysis, this should lead to detection even without freezing/denaturation.

Taking ~8 hrs to complete due to the 12-13 min ramping up and 2-3 min ramping down rates with 5 second holds at 95°C and -10°C, this preliminary experiment was supportive of our hypothesis and further indicated that ~10 freezing/denaturation cycles were optimal for viral RNA release from MS2 coliphage virions (ESI Fig. 6). Thus, faint bands were observed only at 500 GE/reaction at zero freezing/denaturation cycles, with similar levels of detection achieved after 5 cycles with 100 GE/reaction. Interestingly, at 10 cycles, strong bands were observed >100 GE/reaction, with faint bands detected with 10 and 3 GE/reaction, in line with the precept that freezing/denaturation and not osmotic shock was releasing viral RNA from the coliphage virions. However, no bands were detected at 15 cycles. As RNases operate across ambient to 85°C temperatures[48] and RNase removal was not included in the preparation of AR14, we interpreted these results as indicative of RNase-mediated degradation during the protracted manual freezing/denaturing ramping procedure, especially after 10 freezing/denaturation cycles. Indeed, biofluids are very rich in RNases;[49] we therefore reasoned that repeated ramping across the active temperature range of RNases would additionally compromise template integrity and undermine assay performance, even with automated, rapid thermal cycling. Therefore, we substituted 95°C ribonucleoprotein particle denaturation during viral particle disruption with simple thawing.

a)

```
        NP Forward Primer                              NP Probe
        5'-TCTGACATGGATTACCACAAGATC-3'   5'-AGGTCTGTCCGTTCAA-3'
2014:   5'-TCTGACATGGATTACCACAAGATCTTGACAGCAGGTCTGTCCGTTCAACAGGGGATTGTTCGGCAAAGAGTCATCC-3'
1976:   5'-TCTGACATGGATTACCACAAGATCTTGACAGCAGGTCTGTCCGTTCAACAGGGGATTGTTCGGCAAAGAGTCATCC-3'
                                                                     3'-CTAACAAGCCGTTTCTCAGTAGG-5'
                                                                              NP Reverse Primer
      GP Forward Primer                                     GP Probe
       5'-TTTTCAATCCTCAACCGTAAGGC-3'      Dye-5'-GCAGCGATGGGGTGGCACATG-3'-Quencher
1976: 5'-…TTTTCAATCCTCAACCGTAAGGCAATTGATTTCTTGCTGCAGCGATGGGGCGGCACATGCCACATTCTGGGACCGGACTG…-3'
2014: 5'-…TTTTCAATCCTCAACCGTAAGGCAATTGACTTCCTGCTGCAGCGATGGGGTGGCACATGCCACATTTTGGGACCGGACTG-…-3'
                                                                          3'-GTGTAAGACCCTGGCCTGAC-5'
                                                                                GP Reverse Primer
```

b)

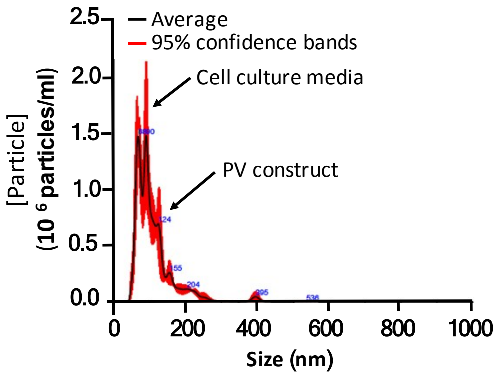

ESI Figure S1: Prediction of qRT-PCR assay performance impact due to genetic differences of Ebola virus Zaire Mayinga (1976) and Makona Guéckédou (2014). (A) Armored RNA ® and HIV1-based pseudotyped viruses were engineered to encode an 154 bp RT-PCR assay target site for the 1976 (PV76) or the 2014 (AR14, PV14) versions of the Ebola virus nucleoprotein (NP) and glycoprotein (GP) genes (NP and GP targets concatenated sequentially 5'→3') as surrogate Ebola virus assay standards. Construct characterisation (PV76) by nanoparticle tracking analysis (C) indicates an estimated PV virion size of ~124 nm, distinct from cell culture supernatant constituents (average and confidence bands of three independent tracking analyses).

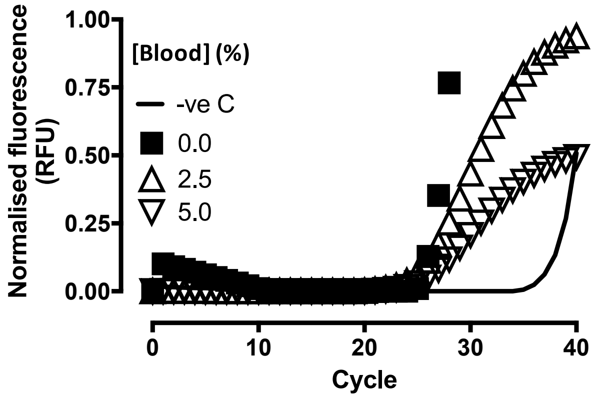

ESI Figure S2: Blood inhibits the fluorogenic signal of 10x Sybr Green but the onset of PCR amplification. Real time PCR reactions for 5.5 x 10^3 copies of lambda phage DNA using 10x Intercalator dye (Sybr-Green 1) in the presence of increasing concentrations of fresh human blood result in comparable amplification onset but blood concentration-dependent inhibition of reaction fluorogenicity. Reaction progression is reported for each of two technical replicates in relative fluorescence units (RFU) normalised to no template control (-ve C) against PCR cycle value.

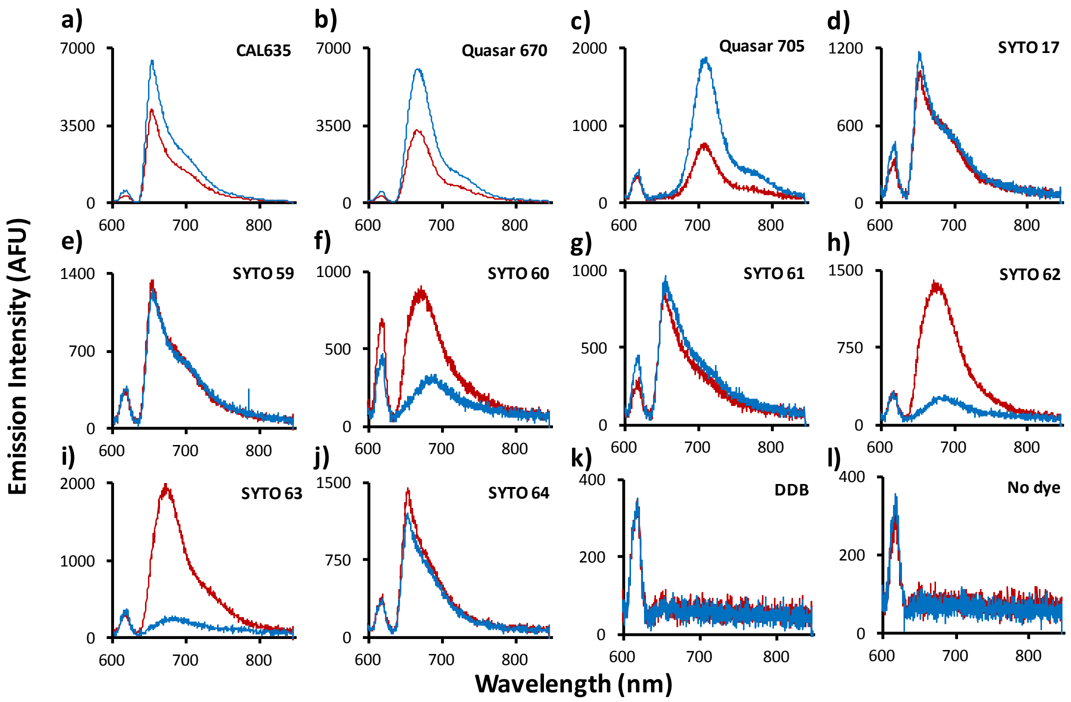

ESI Figure S3: Red / Far red dye fluorescence spectra in the presence and absence of 10% v/v blood. To determine the utility of red/far red fluorescent probes (A-C) and DNA stains (D-J) for qPCR and RT-qPCR in reactions containing blood, the fluorescence spectra of a range of commercially available compounds were determined in presence (red traces) and absence (blue traces) of human blood, with 1,2-Diamino-4,5-dimethoxylbenzene (DDB; k) and no dye measurements used as controls.

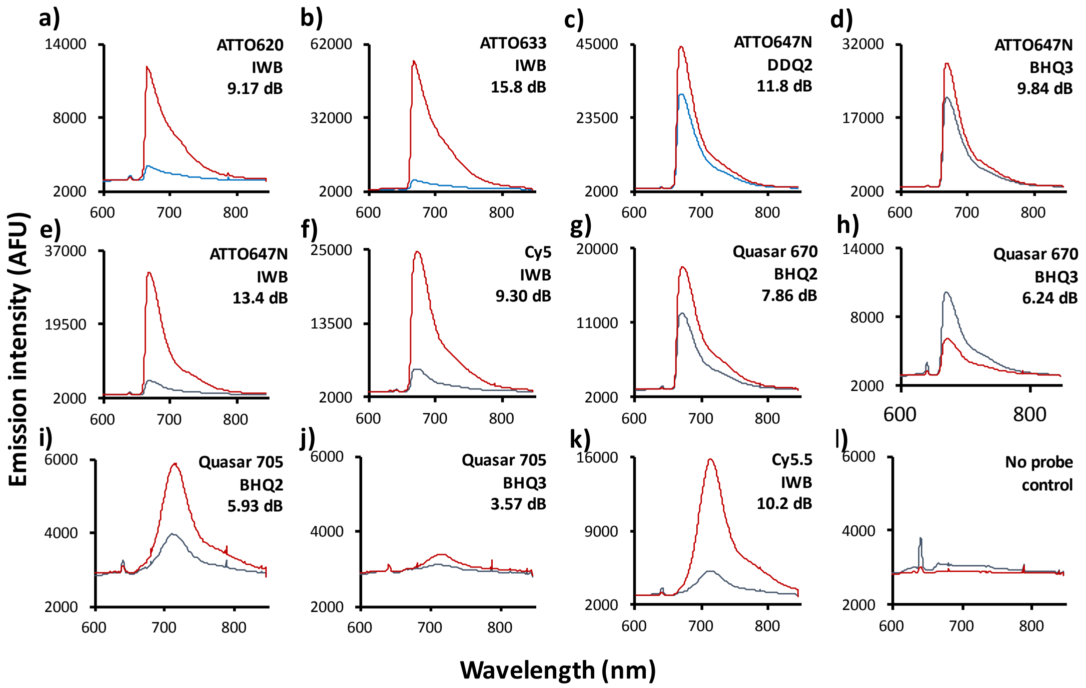

ESI Figure S4: Red / Far red dye-quencher probe fluorescence spectra in the presence and absence of 5% v/v blood. The commercially available red/far red dyes ATTO (A-E), Cy (F, K) and Quasar (G-I) were synthesised on the Trombley EBOV GP probe in combination with Iowa black (IWB; A, B, E, F, K), deep dark quenchers (DDQ;C) or black hole quenchers 2 and 3 (BHQ; D,G-K) to triage blood compatible chemistry selection on the basis of synthesis cost, yield and fluorogenic potential in the presence (red) and absence (blue) of human blood. The signal to noise ratio at the emission peak relative to the blood only control is expressed in decibels (dB) for each probe chemistry.

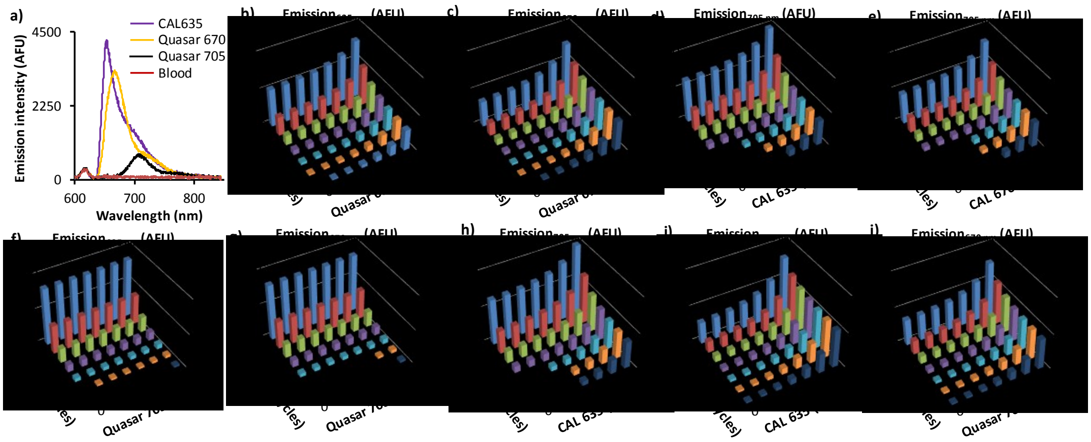

ESI Figure S5: Modelling probe-based RT-qPCR and qPCR multiplexing in whole blood through spectral unmixing. The potential for multiplexing RT-qPCR in the presence of blood was determined through emission spectrum comparison for three red/far red dyes (A), demonstrating adequate peak separation relative to the background fluorescence of 10% v/v blood. Fluorogenic signal deconvolution modelling in the presence of blood for a duplex qPCR with CAL635 and Quasar 670 probes shows reliable signal detection potential at 635 nm (B) and 670 nm (C) from the first amplification cycle of either dye. Use of Quasar 705, however, restricts detection reliability at 705 nm whether CAL635 (D) or Quasar 670 (E) is used as the second dye. Use of Quasar 705 also impacts on amplification detection for the second dye as exemplied by composite signal detection at 635 nm in a CAL635 / Quasar 705 duplex (F) or at 670 nm in a Quasar 670 / Quasar 705 duplex (G), respectively. Triplexing the dyes does not bring composite signal strength above background at 705 nm as illustrated after 2 amplification cycles with CAL635 (H), however the signal exceeds background emission at either 635 nm (I) or 670 nm (J), making target quantification accurate in these two wavelengths, and necessitating full amplification at 705 nm ahead of 635 nm or 670 nm. The emission spectrum monitored in each graph (Y axis)is stated within each panel. Empty columns indicate composite signal levels below the blood background threshold.

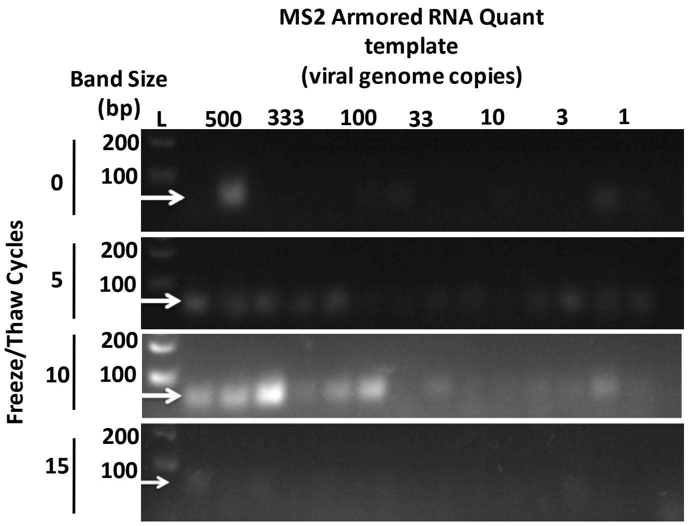

ESI Figure S6: Utility of freezing and boiling in the release of virion genetic material. An acetone/dry ice bath and a dry heating block were used to manually cycle samples of blood-free Armored RNA® QUANT between -20°C and 98°C. The samples were subjected to endpoint one-step RT-PCR using diagnostic grade, pre-validated primer sets and amplification conditions, to evidence amplicon generation by 3% w/v agarose gel electrophoresis against a 100bp DNA ladder (L). Arrows identify the expected ~80bp amplicon size band.

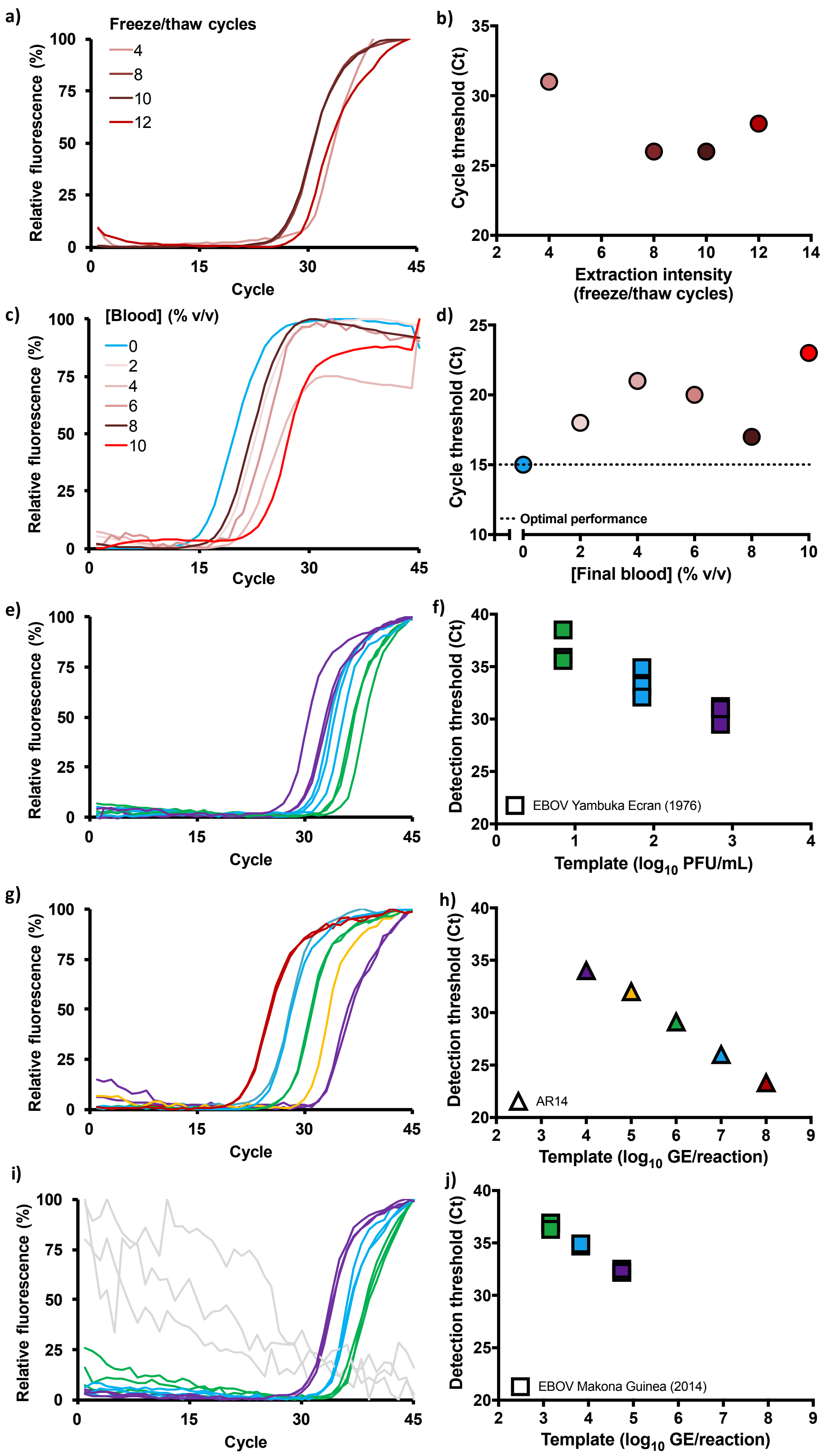
a)
Freeze/thaw cycles
4
8
10
12
Relative fluorescence (%)
Cycle
b)
Cycle threshold (Ct)
Extraction intensity
(freeze/thaw cycles)
c)
[Blood] (% v/v)
0
2
4
6
8
10
Relative fluorescence (%)
Cycle
d)
Cycle threshold (Ct)
Optimal performance
[Final blood] (% v/v)
e)
Relative fluorescence (%)
Cycle
f)
Detection threshold (Ct)
EBOV Yambuka Ecran (1976)
Template (log10 PFU/mL)
g)
Relative fluorescence (%)
Cycle
h)
Detection threshold (Ct)
AR14
Template (log10 GE/reaction)
i)
Relative fluorescence (%)
Cycle
j)
Detection threshold (Ct)
EBOV Makona Guinea (2014)
Template (log10 GE/reaction)

ESI Figure S7. Ct calls and amplification curves for key experiments on the QuRapID platform. The amplification curves (A, C, E, G, I) of experiments depicted in Fig. 5A, 5B, 5D and 5E (reproduced here in B, D, F, and H & J, respectively) illustrate reaction progression on account of incrementally higher levels of freeze-thaw cycling (A, B; 10^5 GE/reaction AR14) or fresh human blood concentration (C, D; 6.6 x 10^8 GE/reaction AR14). These results (A-D) were obtained using electronic pipettes in experiments carried out under BSL2 conditions, and the resulting optimised settings of 10 freeze-thaw cycles and 8% v/v blood were independently tested by separate individuals using manual pipettes under BSL4 conditions (E-J), with foetal bovine serum (FBS) without stabilisation reagents used as a surrogate to fresh human blood at a final reaction concentration of 8% v/v. These experiments included calibration runs using ten-fold serial dilutions of starting at AR14 10^8 GE/reaction (G, H) and analytical runs on EBOV (E, F, I, J) diluted in tissue culture media starting at 700 PFU/ml and spiked with FBS (E, F) or diluted directly in FBS starting at 1.1 x 10^5 PFU/reaction (I, J), as well as assays on other closely related filoviruses not detectable by the Trombley assay (ESI Table S2; negative data not shown). Independent replicates are shown in all panels, with grey lines (E, G, I) illustrating negative control reactions. All replicates in E-J were carried out on independent days whereas experiments in A-D were carried out on the same day. All amplification curves and Ct call points in corresponding panel pairs (AB, CD, etc.) are colour coded for easier data comparison between data presentation formats.

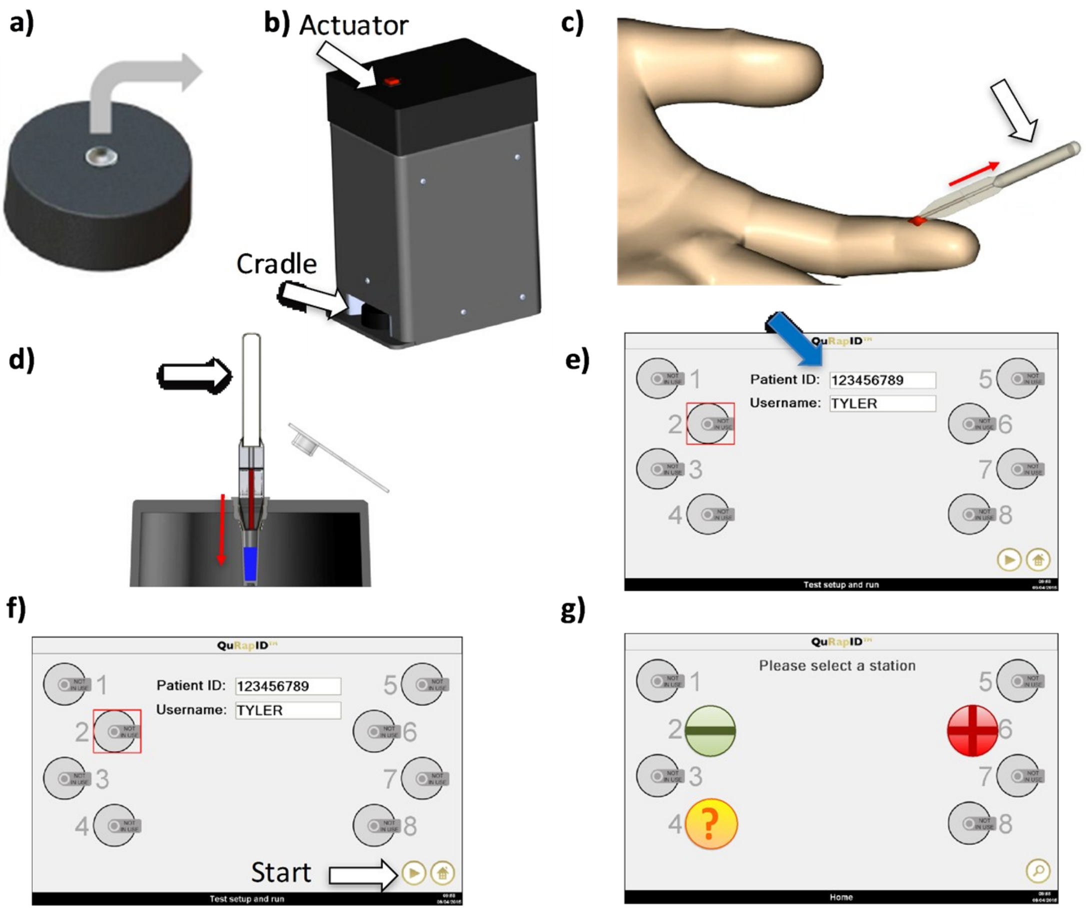

ESI Figure S8: The end-user SOP for QuRapID running a point of need EBOV qRT-PCR. A) Arm the reaction vessel consumable containing the lyophilised reagents by removing the protective film from the top of the puck. B) Place the puck into the puck cradle (blue arrow) in the fixed volume buffer dispenser station and dispense 57.5 ul of reaction buffer by pressing the red button actuator. C) Aspire (red arrow) a fixed volume, 5 ul digital venipuncture (finger capillary) blood sample using a sheathed lancet and fixed volume capillary, by pressing the capillary bulb (white arrow) to withdraw a fixed volume of blood. D) Dispense (red arrow) the blood sample into the consumable by compressing the capillary bulb (white arrow), and seal it with the detachable optical lid. E) Input user and patient ID on the touchscreen keyboard and place the consumable in the automatically pre-selected position highlighted on-screen (red square) F) Lower the corresponding heated sensor lid and press start (blue arrow). G) Read test outcome on-screen and dispose of the consumable as per health and safety regulations. The touchscreen screenshot shows a positive (red '+' circle), negative (green '-' circle) and failed (yellow '?' circle) test example.

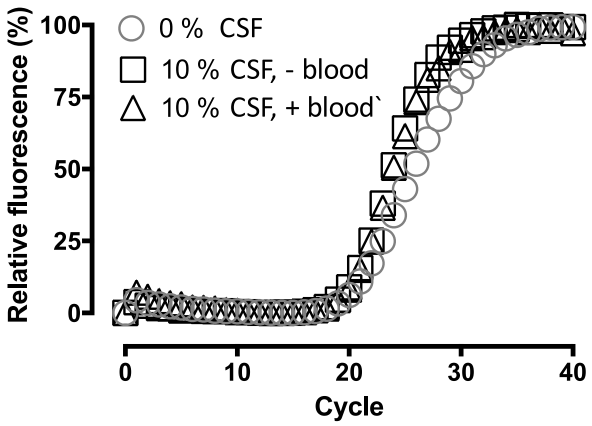

ESI Figure S9: Blood contamination does not affect RNA virion genome detection in cerebrospinal fluid. No significant Ct call difference was observed for 10^8 GE/reaction of AR14 in CSF-free (0%) reactions and reactions containing 10% v/v CSF from two independent subjects with no or high blood contamination during collection.

ESI Table 2: Wild type filovirus and surrogate virus stocks used in this work

Virus	Stock concentration				Containment	
	TCID50/ml*	PFU/ml**	particles/ml	GE/ml***	use level	Accession no.
AR14	-	-	-	1.0×10^{12}	BSL2 / BSL4	KJ660348
PV14	-	-	-	1.0×10^{9}	BSL2	KJ660348
PV76	-	-	7.5×10^{8}	-	BSL2	AF086833
Zaire ebolavirus Yambuka-Ecran (1976)	1.0×10^{7}	7.0×10^{6}	-	-	BSL4	AF086833
Zaire ebolavirus Guinea Makona (2014)	3.0×10^{8}	2.1×10^{8}	-	-	BSL4	KJ660348
Sudan ebolavirus	5.6×10^{7}	3.9×10^{7}	-	-	BSL4	AY729654.1
Bundibugyo ebolavirus	5.6×10^{4}	3.9×10^{4}	-	-	BSL4	FJ217161.1
Taï Forest ebolavirus	1.0×10^{7}	0.7×10^{6}	-	-	BSL4	FJ217162.1
Marburg marbugrvirus (Ravn virus)	3.2×10^{7}	2.2×10^{7}	-	-	BSL4	DQ217792.1

* Actual virus titre
** Estimated virus titre
*** Calibrated on extracts against synthetic RNA

e primer and probe se

Template					robe heterodimer	
					ΔH	ΔS
	(kcal/ml)	(kcal/mol)	(ca/mol/K)	(kcal/ml)	(kcal/mol)	(ca/mol
Ebola virus - Mayinga, Zaire (1976)	-7.17	-89.6	-266	-25.0	-161	-439
Ebola virus - Makona-Gueckedou (2014)	-8.10	-93.0	-274	-21.4	-146	-400

ESI Table 3: Primer and probe sets used in this study.

Organism	Genome Accession	Target Gene	Assay name	Oligonucleotide name	Sequence & modifications	Fina[illegible] concentr[illegible] (nM[illegible]
Ebola virus - Mayinga, Zaire (1976)	AF086833.2	NP	Trombley NP	NP_F	5’ - TCT GAC ATG GAT TAC CAC AAG ATC - 3’	900
				NP_R	5’ - GGA TGA CTC TTT GCC GAA CAA TC - 3’	900
				NP_probe	6FAM - AGG TCT GTC CGT TCA A - MGBNFQ	200
Ebola virus - Mayinga, Zaire (1976)	AF086833.2	GP	Trombley GP	GP76_F	5’ - TTT TCA ATC CTC AAC CGT AAG GC - 3’	1000
				GP76_R	5’ - CAG TCC GGT CCC AGA ATG TG - 3’	1000
				GP76_probe	6FAM - CAT GTG CCG CCC CAT CGC TGC - TAMRA - 3’	100
Ebola virus - Makona-Gueckedou (2014)	KJ660348.2	GP	Trombley +	GP14_F	5’ - TTT TCA ATC CTC AAC CGT AAG GC - 3’	1000
				GP14_R	5’ - CAG TCC GGT CCC AAA ATG TG - 3’	1000
			GP	GP14_probe	6FAM -GCA GCG ATG GGG TGG CAC ATG - BHQ1 - 3’	100
			GP+	GP14_T+probe	5' - Quasar670 -GCA GCG ATG GGG TGG CAC ATG - BHQ2 - 3’	200
Homo sapiens	GRCh37.p8	RPP30	RP	HURNASEP_F	5’ - AGA TTT GGA CCT GCG AGC G - 3’	300
				HURNASEP_R	5’ - GAG CGG CTG TCT CCA CAA GT - 3’	300
				HURNASEP_probe1	6FAM - TTC TGA CCT GAA GGC TCT GCG CG - BHQ1 - 3’	100/2[illegible]
				HURNASEP_probe2	5’- Cy5 - TTC TGA CCT GAA GGC TCT GCG CG - BHQ2 - 3’	100/2[illegible]
Hepatitis C Virus	AB030907	5' ITR	HCV G2b	KY80	5' - GCA GAA AGC GTC TAG CCA TGG CGT - 3'	200
				KY78	5'-CTC GCA AGC ACC CTA TCA GGC AGT ACC AC - 3'	200
Sudan virus	AY729654	GP	PHE SDV GP	F583	5’ - AGG ATG GAG CTT TCT TCC TCT ATG - 3’	650
				R659	5’ - TAC CCC CTC AGC AAA ATT GAC T - 3’	900
				p608SB	6FAM - CAG GCT GGC TTC AAC TGT AAT TTA CAG AGG - BHQ1	250
Bundibuyo virus	FJ217161	NP	PHE SDV NP	F2016	5’ - ATG GAA ACC AAG GCG AAA CTG - 3’	900
				R2089	5’ - TAC TTG TGG CAT TGG CTT GTC T - 3’	900
				p2045S	6FAM - CGG GTA GCC CCC AAC - MGBNFQ	250
Taï Forest virus	FJ217162	NP	PHE TF NP	F360	5’ - TTT GCC GTG CAT TTA GAA TAA GAG - 3’	900
				R438	5’ - CTG CCG GGT CTG GTG AGA T - 3’	650
				p385KSR	6FAM - CAA CTT AAA CCT CCG ATT CGG CAA CAC A - BHQ1	250
Ravn virus	EF446131	NP	PHE RAVN NP	F1190	5’ - CTC CAG AAG ACT GAG ATT ACA CAC AGT - 3’	650
				R1269	5’ - GCA GCA AGA CGG GCT AGT TT - 3’	650
				P1218KSR	6FAM - AGA CAT TGG CCG TCC TCA GCC AGA A - BHQ1	250
